# Coupling Mechanical Deformations and Planar Cell Polarity to Create Regular Patterns in the Zebrafish Retina

**DOI:** 10.1371/journal.pcbi.1002618

**Published:** 2012-08-23

**Authors:** Guillaume Salbreux, Linda K. Barthel, Pamela A. Raymond, David K. Lubensky

**Affiliations:** 1 Department of Physics, University of Michigan, Ann Arbor, Michigan, United States of America; 2 Max Planck Institute for the Physics of Complex Systems, Dresden, Germany; 3 Department of Molecular, Cellular, and Developmental Biology, University of Michigan, Ann Arbor, Michigan, United States of America; Princeton University, United States of America

## Abstract

The orderly packing and precise arrangement of epithelial cells is essential to the functioning of many tissues, and refinement of this packing during development is a central theme in animal morphogenesis. The mechanisms that determine epithelial cell shape and position, however, remain incompletely understood. Here, we investigate these mechanisms in a striking example of planar order in a vertebrate epithelium: The periodic, almost crystalline distribution of cone photoreceptors in the adult teleost fish retina. Based on observations of the emergence of photoreceptor packing near the retinal margin, we propose a mathematical model in which ordered columns of cells form as a result of coupling between planar cell polarity (PCP) and anisotropic tissue-scale mechanical stresses. This model recapitulates many observed features of cone photoreceptor organization during retinal growth and regeneration. Consistent with the model's predictions, we report a planar-polarized distribution of Crumbs2a protein in cone photoreceptors in both unperturbed and regenerated tissue. We further show that the pattern perturbations predicted by the model to occur if the imposed stresses become isotropic closely resemble defects in the cone pattern in zebrafish *lrp2* mutants, in which intraocular pressure is increased, resulting in altered mechanical stress and ocular enlargement. Evidence of interactions linking PCP, cell shape, and mechanical stresses has recently emerged in a number of systems, several of which show signs of columnar cell packing akin to that described here. Our results may hence have broader relevance for the organization of cells in epithelia. Whereas earlier models have allowed only for unidirectional influences between PCP and cell mechanics, the simple, phenomenological framework that we introduce here can encompass a broad range of bidirectional feedback interactions among planar polarity, shape, and stresses; our model thus represents a conceptual framework that can address many questions of importance to morphogenesis.

## Author Summary

Many tissues and organs, including sensory organs like the vertebrate retina and inner ear, are built from sheets of connected cells called epithelia. The precise arrangement of different types of cells within these epithelia can be essential to their function. (For example, photoreceptor cells in eyes must be properly spaced to collect an optimal, undistorted signal.) We combine experimental observations with computational modeling to understand how a particular example of such epithelial organization—the planar crystalline packing of cone photoreceptor cells in the fish retina—is created. Specifically, we introduce a model where the strength of cell-cell adhesion along an interface depends on the orientation of that interface. When a global mechanical compression is applied along one direction, this model can recapitulate observed features of the cone packing and gives qualitatively correct predictions of the cone photoreceptor pattern observed in regenerated and mutant retinas. Our analysis shows that simple local interactions can direct the creation of regular, long-ranged order among epithelial cells, and it also clarifies the mechanical interactions needed to establish and maintain the integrity of the retinal epithelium. Our model may thus ultimately provide a foundation for insights into diseases in which epithelial integrity is lost.

## Introduction

Epithelia are one of the basic building blocks from which animals sculpt complex tissues and organs during development [1]–[5]. These sheets of cells are held together by specialized structures—notably apical junctional complexes, including adherens junctions—that allow cells to adhere tightly to their neighbors and ensure the epithelium's mechanical integrity [6]–[9]. In most epithelia, individual cells of distinct identities are packed together in quasi-two-dimensional arrays of varying complexity. Despite the fundamental importance of epithelial organization for many biological functions, the biophysical mechanisms that control cell shape and position in epithelia—and in particular the development of regular, ordered epithelial cell packings—remain only partially understood.

In vertebrates, the neural retina exhibits a particularly high degree of epithelial organization, both in the radial direction, where it comprises multiple, stratified layers, and within layers, where the spatial distribution of each class of neuron within the epithelial plane has consistently been shown to be non-random [10]. This planar order is especially pronounced in adult teleost fish, where the cone photoreceptor cells are arranged in a well-defined, periodic pattern—the cone mosaic—that shows strong heterotypic as well as homotypic correlations [11]–[12]. The cone mosaic thus represents a rare vertebrate example of the precise regulation of cell fate and organization at the single cell level (more instances of which have been described in invertebrate systems [13]–[14]).

**Figure 1:**
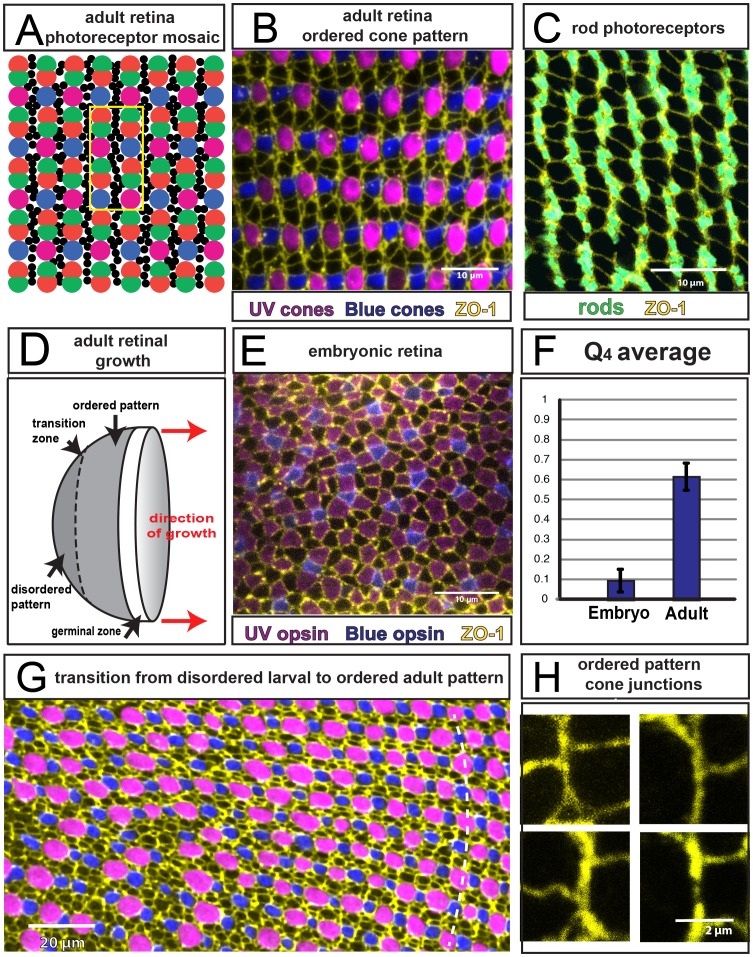
Cone mosaics in the embryonic, larval, and adult zebrafish retina. **A**) Schematic of photoreceptor packing in the apical plane of the adult retina, showing cones of red, green, blue, and UV (magenta) spectral sensitivities, and smaller rods (black). The 12-cell repeating motif of the cone mosaic pattern is outlined by the yellow rectangle. **B**) Regular pattern of cones visualized in a flat-mount retinal preparation from an adult, double transgenic zebrafish (*mi2009* line) in which UV and blue cones express different fluorescent reporters (pseudocolored magenta and blue) under the control of UV and blue opsin promoters, respectively. Apical boundaries of cells are delineated by ZO-1 immunostaining (yellow). Horizontal rows of alternating blue and UV cones alternate with horizontal rows of unlabeled cone profiles representing red-green double cone pairs. **C**) Rod photoreceptors are visualized in a flat-mount retinal preparation from an adult transgenic zebrafish (*kj2* line) in which the rod opsin promoter drives expression of a reporter gene (pseudocolored cyan). Apical boundaries of cells are delimited by ZO-1 immunostaining (yellow). Rods are largely excluded from the vertical columns of contiguous cones, and instead occupy the spaces between adjacent columns. **D**) Schematic of the hemispherical retinal epithelium. Retinal neurogenesis in the circumferential germinal zone at the peripheral margin adds annuli of new retinal neurons, such that the age of retinal cells is a direct function of their distance from the periphery. The ordered cone pattern illustrated in panels A–C is in the peripheral retina, whereas the central retina, surrounding the pole of the hemisphere, exhibits the disordered embryonic/larval pattern (panel E), with a transition zone in-between (panel G). The annular ligament (not shown) roughly encircles the germinal zone. **E**) Apical retinal surface of a double transgenic *mi2009* zebrafish at 4 days post-fertilization, showing the packing of cone cells that differentiated during embryonic development, before progressive addition of cones at the retinal margin begins: UV cones are magenta, blue cones are blue, ZO-1 immunostaining (yellow) outlines cell profiles at the level of the OLM. **F**) Average orientational order parameter 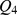 (Fig. 2 and Methods) for embryonic and adult retina. (Three and six regions of ∼20 by 15 cone cells were used to calculate the values for embryonic and adult retina, respectively.) **G**) Transition from disordered cell packing in the larval remnant (left side) to ordered packing (right side) in a flat-mount retina of an adult double transgenic zebrafish, *mi2009* labeling blue and UV (magenta) cones, with cell boundaries visualized with ZO-1 immunostaining (yellow). The curved, dashed line segment traces a cone column. **H**) High magnification views of the angles at which three cone-cone interfaces meet (ZO-1 in yellow).

Previous studies have characterized cone mosaic patterns primarily by observing regular spatial arrangements of various individual cone cell subtypes, identified morphologically and/or with specific cell markers [15]–[18]. They have, in contrast, left largely unexplored the complementary question of how cone photoreceptors, together with rod photoreceptors and the apical processes of Müller glia, pack together and occupy space in the epithelial plane. Depending on the species, cone photoreceptors in teleost fish include several morphologically identifiable classes of single cones and double cones that express distinct visual pigments [19]–[20]. For example, the zebrafish, *Danio rerio*, has four spectral subtypes of cones designated red, green, blue, and ultraviolet (UV), respectively, based on the absorption maxima of their visual pigments [21]–[22]. These cone photoreceptors are distributed in a repeating pattern that has been classically described as a row mosaic (Fig. 1) [11], [15], [17], [23].

The zebrafish retina is a thin, hemispheric sheet that lines the back of the eye. This sheet continues to grow along with the rest of the fish throughout postembryonic larval and adult stages: the diameter of the eye at the end of embryonic development (∼3 days post-fertilization [dpf]) is only ∼0.2 mm, but several months later it can reach ∼2 mm or more. From ∼3 dpf onwards, retinal growth is accomplished by the addition of new cone cells at the rim of the retinal hemisphere, where the retinal and ciliary epithelia meet at a circumferential germinal zone of proliferative precursor cells (Fig. 1) [24]–[27]. Due to this particular mode of continuous growth, successive stages of development and cell differentiation are laid out spatially in concentric annuli in a single epithelium: the remnant of the embryonic and larval retina remains in the center of the retina of the adult fish, whereas the majority of the adult retina extending out to the periphery is more recently created tissue [15]. The embryonic/larval remnant is easily distinguished in a flat-mounted preparation of the entire adult retina because neither the cones generated from the embryonic retinal primordium nor those added post-embryonically to the growing larval retina are arrayed in a regular, rectangular mosaic [15], [18]. Even though the growing retina adds annuli of new cones at the periphery from late embryonic stages onwards, only those born after the end of larval development, at ∼3 weeks post-fertilization (wpf), form an ordered mosaic lattice. Thus, addition of successive annuli of cone photoreceptors at the retinal perimeter is not, by itself, sufficient to produce a crystalline cone mosaic.

The appearance of the ordered lattice of cone photoreceptors at ∼3 wpf, on the other hand, *does* coincide with the completion of significant developmental changes in ocular anatomy. These include the formation and maturation of the anterior segment—that is, the iris, the ciliary epithelium, and the annular ligament, a circular bracket of connective tissue that is thought to give structural support to the front of the eye and that roughly encircles the retinal germinal zone [28]. The maturation of the anterior segment leads to the production of aqueous humor, a fluid secreted by the ciliary epithelium that fills the eyeball. The aqueous humor is maintained at a significant intraocular hydrostatic pressure (IOP) relative to the outside environment, and this pressure inflates and stretches the retinal epithelium [29]–[31]. Similar mechanical stresses are known to affect epithelial cell packing in other contexts, but the potential relationship between these tissue-scale influences and the organization of the cone mosaic pattern has not been explored.

Another mechanism known to influence cell shape and packing in epithelia is planar cell polarity (PCP)—the organization of cellular properties along a preferred direction within the plane of an epithelium [32]–[33]. Such polarization is increasingly recognized as a widespread and important feature of epithelial organization. PCP has not previously been studied in the vertebrate retina, but its molecular mechanisms have been worked out in considerable detail in certain *Drosophila* model systems [34], and the same pathway appears to be conserved in some vertebrate systems [35]. One of the major functions of PCP is to introduce anisotropic mechanical stresses in epithelial sheets through modulation of acto-myosin cortical contractility or cell-cell adhesion, leading to polarized cell shape changes and rearrangements [34], [36]; conversely, PCP can itself be affected by changes in cell shape and packing [37]–[38] and by mechanical stress [39]. Several mathematical models of PCP have been developed, ranging from the relatively molecularly detailed to the more schematic and phenomenological [37]–[38], [40]–[46], and the consequences of polarized contractility and adhesion for cell movement have also been examined computationally [47]–[49]. A mathematical model that can capture the full range of interactions between PCP and mechanical forces has, however, so far been lacking.

Here, we propose just such a model to explain the developmental mechanisms behind the emergence of the ordered cone mosaic in the adult zebrafish retina. We present the first systematic experimental characterization of the epithelial packing of cone and rod photoreceptors and Müller glia at the apical surface of the retina, and we describe both the evolution of packing order as new cells are generated during retinal growth and the defects in order that accompany photoreceptor regeneration and that occur in a mutant strain of zebrafish with elevated intraocular pressure. Based on our observations, we introduce a mathematical model in which anisotropic, tissue-scale mechanical stresses interact with intrinsic planar cell polarity (PCP) in cones to generate cell packing in a rectangular lattice with long-ranged order. We provide morphological observations to verify the existence of the postulated PCP and functional genetic data consistent with the predicted role of anisotropic mechanical stress in the generation of the rectangular cone lattice.

## Results

### A rectangular cone cell lattice is a property of the adult but not of the larval or embryonic retina

We used immunostaining against the apical junction protein, Zonula Occludens-1 (ZO-1), which labels the apical cell profiles at the level of the outer limiting membrane (OLM) in the vertebrate retina [50], in order to image the precise cell boundaries and cell arrangements within the photoreceptor cell packing at various stages of retinal growth. To identify the cells whose apical profiles are delimited by ZO-1 we used several transgenic zebrafish lines in which fluorescent reporters (enhanced green fluorescent protein, EGFP, or a monomeric red fluorescent protein, mCherry) are driven by cell-specific promoter sequences: *sws1* (ultraviolet opsin) for ultraviolet cones [51], *sws2* (blue opsin) for blue cones [21]; cone alpha-transducin for all cones [52], *rh1* (rod opsin) for rod photoreceptors [53]; and *gfap* (glial fibrillary acidic protein) for Müller glia [25].

We first characterized the apical epithelial organization in the photoreceptor layer of adult zebrafish. The photoreceptor mosaic pattern in zebrafish has been previously described by observing the positions of photoreceptor cells in flat-mount retinal preparations [15]–[17], [23]. Cones in the adult retina are organized in a rectangular lattice consisting of a repeated motif of 12 cells with an internal, reiterative, mirror image symmetry (Fig. 1A). Rows of blue and UV single cones alternate with rows of red and green double cone pairs (Figs. 1A–B; S1A–C). Double cone pairs are tightly apposed along the length of their apical processes (inner segments) [17], [54]. In the orthogonal direction, columns of cones can be separated by rods, which have much smaller profiles (Figs. 1A, C; S1D–I). The adult teleost retina continues to grow by addition of retinal cells in a circumferential germinal zone at the retinal periphery (Fig. 1D), and each column of cones (Fig. 1A) represents a cohort of cells that are generated synchronously [15], [55] and differentiate sequentially [55]. Rods appear after cones differentiate; they continue to accumulate in the adult retina and insert into the epithelial sheet between cone columns (Fig. S1D–I). The earliest born rod photoreceptors insert into the cone pattern at the corners defined by the four-way interface of blue, UV, red, and green cones (Fig. S1D–F; [16]); as the fish ages, rods also accumulate elsewhere between the cone columns (Fig. S1G–I). Rod photoreceptors have been shown not to be essential for generation of the cone mosaic in goldfish retina [56], and we subsequently ignore rods in our analysis of cone cell packing. Finally, the numerous, irregular ZO-1-delimited cell profiles at the apical surface of the retina in the germinal zone and in the region of differentiating cones at the peripheral margin are processes of Müller glial cells, which have thin lamellae that completely enwrap the cone and rod photoreceptors as they penetrate through the OLM (Fig. S1J–L).

Labeling the cell boundaries with ZO-1 antibodies reveals additional, unexpected details of the apical epithelial packing and shape of cones at the level of the OLM. The two orthogonal directions in cone packing geometry are not equivalent: boundaries between adjacent cones belonging to the same column are straight, particularly the junctions between pairs of red and green double cones within a column (Figs. 1B–C; S1B, E, H). These columns of cones belong to a cohort generated synchronously at the germinal zone, and they remain contiguous; rods penetrate the cone lattice between the columns but rarely between cones within the same column (Figs. 1C; S1G–I). The nonequivalence in the packing geometry of cones is suggestive of a cell-cell adhesion mechanism that operates between cones within a column but not across columns.

In contrast to those in the adult retina, cones that differentiate within the first few weeks after fertilization are not organized into long-range, supracellular lines (Fig. 1E, G), and their packing clearly lacks the periodic, repetitive, lattice organization of the adult retina. Although short, linear arrays of alternating blue and UV cones are apparent even in the embryonic remnant (Fig. 1E), they are not aligned in a consistent direction and red-green double cone pairs cannot be recognized at this stage [54]. The embryonic and early larval retina is also known to have relatively more blue and UV cones and relatively few rods compared with the adult retina [15]. In the larval retina, which is formed by addition of cells at the retinal margin (lower left, Fig. 1G), the ordered linear fragments become more prominent, but the long-ranged lattice order is still clearly absent.

**Figure 2:**
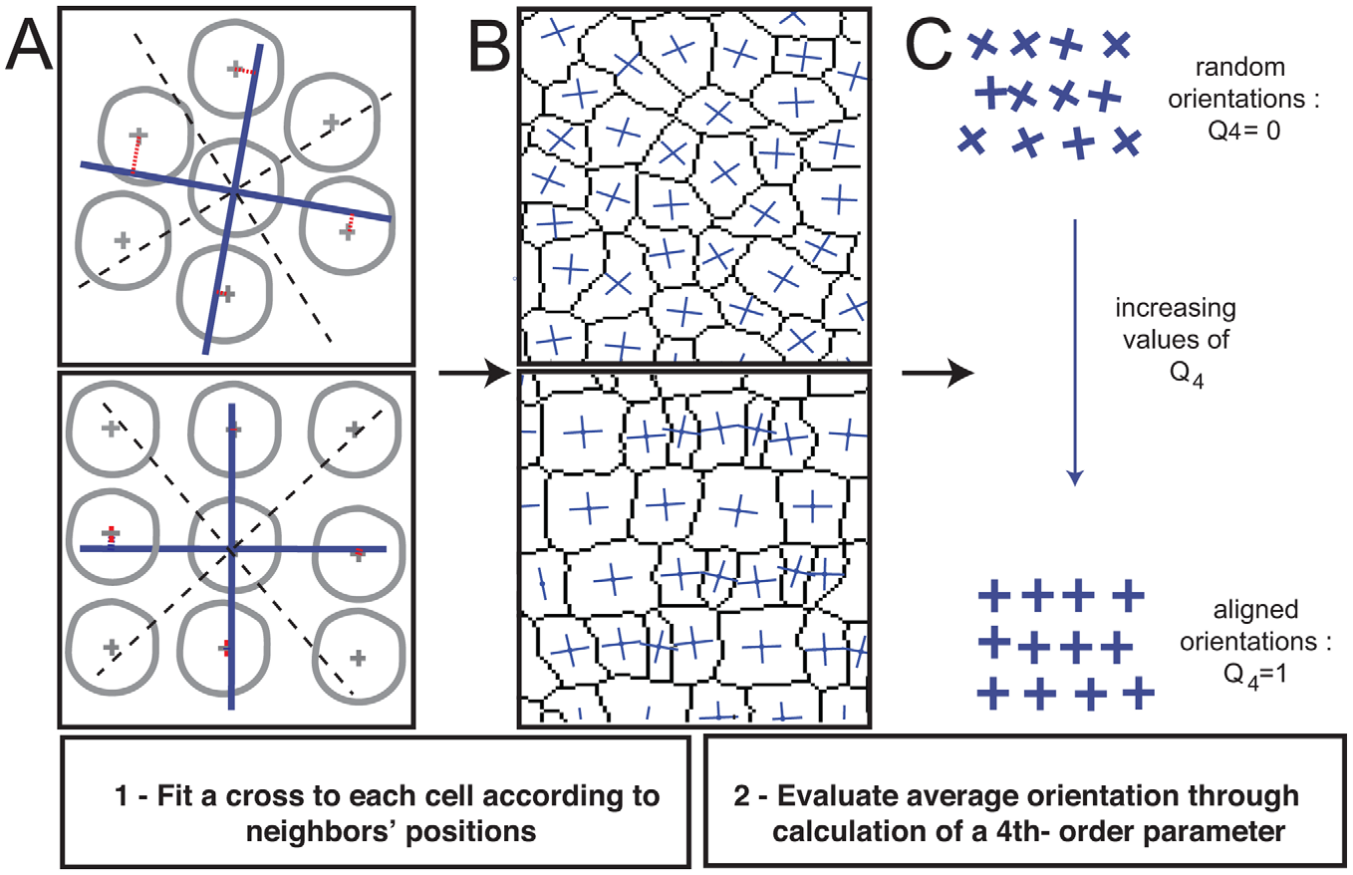
Defining the orientational order parameter *Q*_4_. Top two panels of A and B represent disordered cell packing; the bottom two panels of A and B represent ordered cell packing. **A**) A cross (blue) is placed at the geometric center of each cell profile, and the local fourfold orientation is chosen by minimizing the mean square distance between neighboring cells and the arms of the cross (red lines). One neighboring cell is chosen in each quadrant (dashed lines). **B**) The orientation of the crosses varies less in an ordered packing. **C**) The magnitude of the fourth order parameter 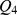 ranges from 0 to 1 and increases as variability in the orientation of the crosses decreases. (See also Methods.)

To obtain a quantitative measure of the regularity of cone cell packing at the level of the apical epithelial surface, we segmented the images of ZO-1 immunolabeled cell profiles and statistically analyzed the data with an orientational order parameter, 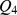, that we designed to measure the similarity of the observed packing to an ideal rectangular lattice (Fig. 2, Text S1, and Fig. S2). Compared to a traditional Fourier transform measurement of positional order, 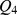 is expected to be more sensitive to relatively weak ordering [57]. It also has the advantage that it measures order relatively locally, without the requirement of averaging over a large number of cells, and thus can detect abrupt changes in the degree of ordering such as that observed at the retinal margin (below). Cones of all four subtypes are treated equivalently in the analysis, so that the value of 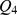 reflects the packing organization only, independent of the distribution of spectral subtypes. The average value of the 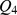 order parameter is significantly different between the embryonic and adult retinas (Fig. 1F): 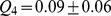 (mean 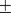 SD, 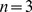) for embryonic retina, and 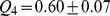 (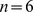) for adult retina. 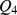 is designed to vanish in a truly disordered packing, though finite size effects always give at least a small positive value when it is calculated from real data; the value for embryonic retina thus indicates that there is little or no orientational order. As an alternative measure of crystalline organization, we also computed a conventional positional order parameter 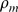 from the Fourier transform of the cell centroid positions and found a similar discrepancy between embryonic and adult retinas (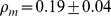 for embryonic retina, 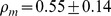 for adult retina). This difference in 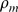 is consistent with that observed in simulations of the liquid-solid transition [58]; in particular, 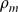 is not expected to vanish in a disordered, liquid-like packing. (See Text S1 and Fig. S3 for details.)

The transition to a rectangular lattice pattern of cone cell packing occurs at the end of larval development, at approximately three weeks of age [28]. The boundary between the larval remnant and the ordered lattice pattern can be visualized in the adult retina (Fig. 1G); the transition in packing order occurs abruptly, over the scale of a few cones.

### Rectangular cone packing emerges from unpatterned neuroepithelium at the germinal zone

**Figure 3:**
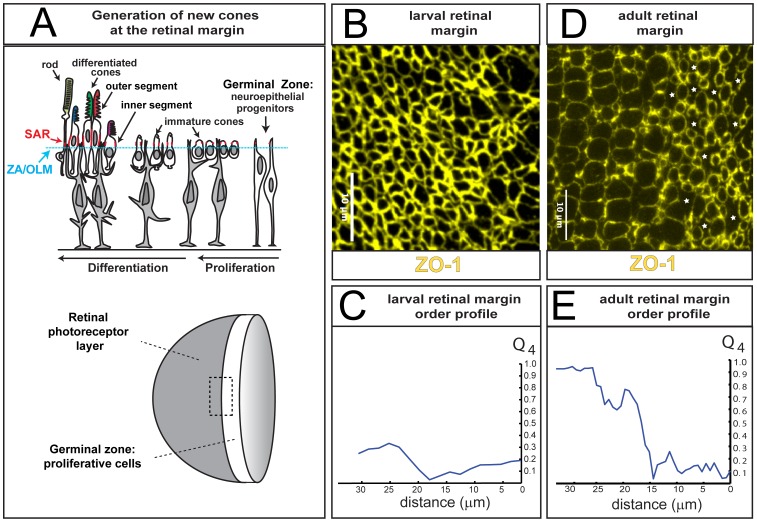
Generation of cone photoreceptors in larval and adult fish. **A**) Top: developmental time is indicated from right to left. Neuroepithelial progenitor cells in the retinal germinal zone proliferate, exit the cell cycle, and differentiate into cone photoreceptors, Müller glia (shaded grey), and retinal neurons (not shown). Rod photoreceptors are later added to the differentiated retina (left). The apical epithelial surface of the retina (cyan line) is the outer limiting membrane (OLM), as defined by the zonula adherens (ZA). The junctional protein Zonula Occludens (ZO-1) localizes to the OLM. The subapical region (SAR) of the plasma membrane shown in red is in the inner segment of the photoreceptors and is the site of localization of the Crumbs complex. The outer segments of photoreceptors contain the rod and cone opsins and are colored to represent the wavelength absorption maxima of their respective visual pigments. Bottom: the dotted rectangle indicates the retinal margin region illustrated in panels B and D, which straddles the proliferative germinal zone and the adjacent zone of differentiating cones. **B**) Larval retinal margin with cell boundaries at the level of the zonula adherens indicated by ZO-1 immunostaining, showing the packing of cone cells added through growth at the margin during the larval stage. The germinal zone is at the right. **C**) The Q_4_ orientational order parameter from the image in panel B is plotted as a function of distance from the proliferating germinal zone (at ∼15 µm). **D**) Adult retinal margin; note that straight vertical columns of cones appear abruptly at the edge of the germinal zone and represent a cohort of cells generated approximately synchronously from the germinal zone at the right. The polygonal profiles marked by white stars represent profiles of Müller glia (see Fig. S1J, L). **E**) The 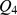 orientational order parameter from the image in panel C; note that the value of 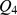 increases sharply at the edge of the germinal zone (at ∼15 µm) in the adult retina.

In order to gain additional insight into the mechanism of cone mosaic formation, we examined the establishment of the cone pattern at the periphery of the retina, where successive cohorts of cone columns are generated in the germinal zone (Figs. 1D, 3A). The spindle-shaped neuroepithelial cells in the germinal zone span the width of the retinal epithelium and form a continuous epithelial sheet with the retinal cells that extend to the apical surface of the differentiated retina, including Müller glia and photoreceptors–rods and cones (Fig. 3A). Rods and cones have an elaborate, cilia-derived extension of their apical surface (Figs. 3A; S4A–F), which includes an inner segment with abundant mitochondria and an outer segment that contains the phototransduction machinery [59].

The apical epithelial cell packing at the boundary between peripheral retina and germinal zone, where newly generated cones are differentiating, shows striking differences between larvae and adults: in the larval retinal margin there is no obvious distinction between the proliferative germinal zone and the differentiated retina, but instead a gradual transition from a region with heterogeneous cell shapes including large, irregular profiles of Müller glia, to the differentiated region where polygonal cone profiles dominate (Fig. 3B). In contrast, a steep transition is clearly visible in the cell profiles in the adult retinal margin from the disordered, heterogeneous proliferative region compared with the crystalline ordered regions of the differentiated retina (Fig. 3D). These distinctions were confirmed by evaluation of the 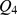 order parameter profile along the direction of growth (Fig. 3C and 3E, respectively). To obtain the value of the 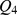 order parameter, we did not consider the polygonal, non-convex ZO-1 profiles that are frequently observed in the adult germinal zone, which are filled by processes of Müller cells (Figs. 3D; S1J–L). These profiles were subsequently removed from our analysis based on a convexity index measurement of segmented zones (Text S1).

### A mathematical model coupling planar polarization and mechanical distortion

#### Motivation

The creation of a crystalline cone mosaic entails at least two distinct processes: The cone photoreceptors must line up in regular columns, and spectral fates must be assigned to cones in the correct ratios. Our focus here is firmly on the former set of events, and our model is thus fundamentally a description of the forces that determine cell shape and position.

Several facts from the preceding sections (Figs. 1, 3) motivate the precise form of this description. First, cone photoreceptors are progressively added at the retinal margin in larval as well as in adult fish. This growth mode thus cannot by itself be sufficient to create a regular cell packing. Instead, the ordered adult pattern is first found at roughly the same developmental time that the anterior segment of the zebrafish eye attains its mature morphology, at the end of larval development [28]. These anatomical and developmental peculiarities lead us to hypothesize that the formation of the annular ligament, by introducing a rigid frame around the retinal margin of the adult eye, induces anisotropic mechanical stresses in the retinal epithelium, with the anisotropy likely to be most pronounced nearest to the annular ligament, which is immediately external to the retinal germinal zone [28]. Cells in the germinal zone tend on average to be mildly elongated in the circumferential direction (Fig. 3D), parallel to the margin, suggesting, more specifically, that the annular ligament may compress the germinal zone in the radial direction (or, equivalently, may protect it from radial tensile stresses present elsewhere in the epithelium).

A second notable fact is that cone photoreceptors in the adult retina align in circumferential columns a single cell wide and parallel to the germinal zone (Fig. 3D). That these annular columns of cones do not wander even on large scales suggests that the junctions between columns are under a relatively high tension that tends to pull the columns straight. This idea is further supported by the observation that, when three cone-cone interfaces meet at a vertex, the angles between the junctions deviate substantially from 120° (Fig. 1H). In mechanical equilibrium, the forces exerted on the vertex by the three interfaces must balance; if the angles between the interfaces are not equal, then some interfaces must be exerting a larger force—and thus have a higher tension—than others. Indeed, from the fact that the two inter-column interfaces meeting at a vertex are nearly parallel, we can infer that their tensions are large compared with that of the third, intra-column interface.

**Figure 4:**
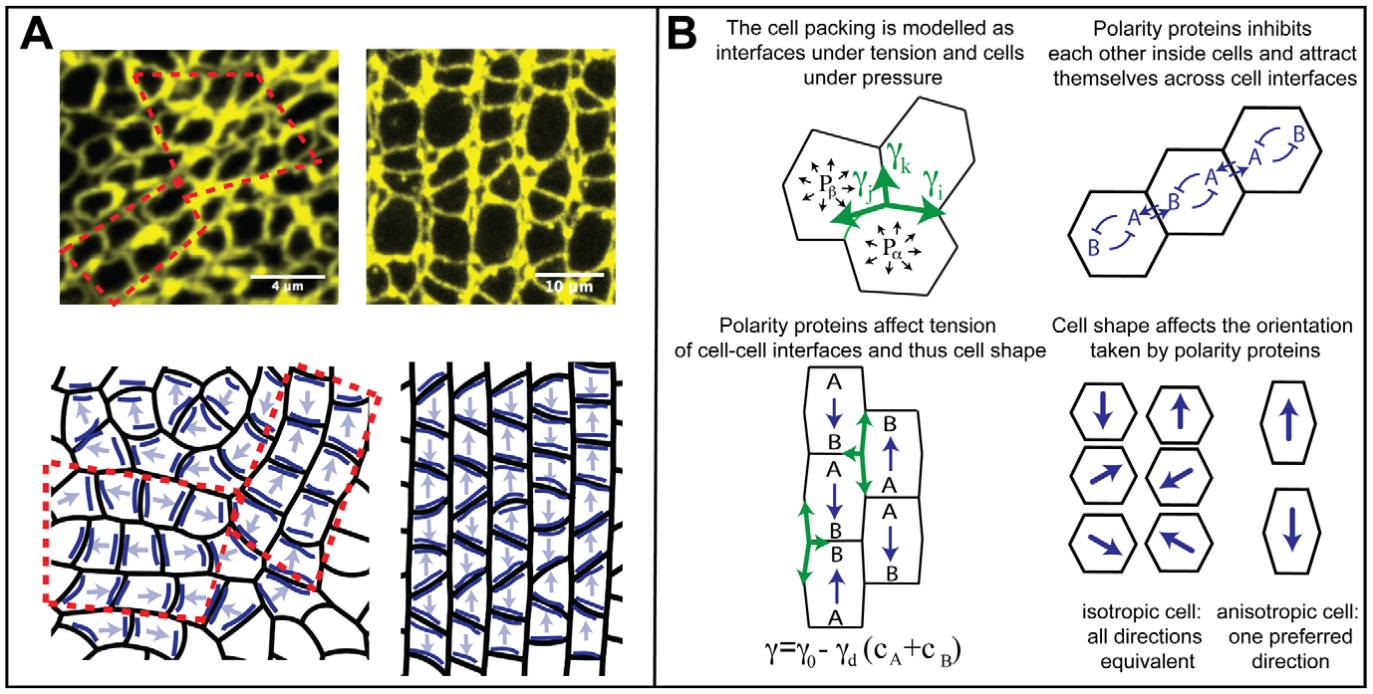
Model rationale and main ingredients. **A**) Cell boundaries at the level of the zonula adherens of the larval retina are revealed by ZO-1 immunostaining; note the fragments of straight, aligned rows of cones (top left, red dashed lines). We propose that this organization reflects an underlying planar cell polarity (schematic, bottom left): polarity proteins (dark blue lines) accumulate on certain interfaces, lowering their tension and leading to cell polarization (arrows). Without a global ordering signal, domains of aligned cell polarity (red dashed lines) appear. In the presence of a global ordering signal, all cones polarize in the same direction (bottom right) leading to the observed rectangular lattice and columns of cones in the adult retina (top right). **B**) Model ingredients: Cell shape is determined by interfacial tensions 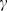 and pressures 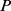; tensions must balance at vertices in mechanical equilibrium (green arrows, top left). Proteins A and B define planar cell polarity (top right) and prefer to collect on shorter interfaces (bottom right). Interfacial tensions depend on polarity protein concentrations *c* (bottom left).

That rod photoreceptors insert primarily between columns of cones is also consistent with the idea that the interfaces between adjacent cone columns are under high tension or have weak adhesion (which amount to the same thing at a coarse-grained level), thus favoring rod insertion. Moreover, the tendency to form columns of cones a single cell wide can be seen not just in the adult retina, but also in the larval remnant, where the columns are not globally aligned, but fragments of columns are nonetheless clearly apparent (Figs. 1G, 3B, and 4A). This indicates that the tendency towards anisotropic cell-cell interactions is not a property exclusively of the ordered adult cone mosaic, but is rather intrinsic to the cone photoreceptors themselves. We thus propose that the cone photoreceptors in the retinal epithelium exhibit polarized cell-cell adhesion and cortical tension mediated by PCP, and that these lead to a tension on cell-cell interfaces that depends on their orientation relative to the direction of planar polarization.

#### Model ingredients

In the absence of a detailed molecular understanding of the determinants of photoreceptor shape and packing in the retina, we turned to a coarse-grained, phenomenological description to understand the essential consequences of our proposed coupling between mechanical forces and PCP. In keeping with earlier successful work on epithelial cell packing [60]–[65], our model focuses on cells' two-dimensional shape at the level of the adherens junctions in the OLM (Fig. 3A). Within this two dimensional model, cells are bounded by *edges*, corresponding to cell-cell junctions, which meet in *vertices* where three or more cells come together. Our model assumes that the edges are circular arcs, as they must be in mechanical equilibrium if the cells possess an isotropic pressure conjugate to their (two-dimensional) area and the cell-cell interfaces do not resist bending [66]; more generally, this assumption represents the simplest possible extension of the widely-used vertex models, which enforce straight edges [37], [60], [62], [67], to allow for the significant interfacial curvature seen in some of our images of fish retina. In addition to the positions of the vertices, the radii of curvature of the edges are dynamical variables that we follow during our simulations.

The model also includes a schematic description of PCP loosely based on what is known about *Drosophila* wing imaginal discs, but flexible enough to allow for alternative molecular mechanisms that also lead to polarization [37], [41]. (It is perhaps worth noting that, although the signaling pathways responsible for PCP in wing discs have been analyzed extensively, genetic studies show that the molecular origins of PCP in some other *Drosophila* tissues, while likely related, cannot be identical [68].) In our model, membrane-bound protein complexes A and B interact in such a way that they prefer to be on opposite sides of a given cell, thereby defining a polarization vector within each cell (Fig. 4B). The extracellular domains of proteins on apposing surfaces in neighboring cells then bind, thus aligning the polarizations of neighboring cells. Although strictly polar order would demand that A complexes in one cell bind only to B complexes on apposing surfaces, we also consider the possibility of interactions reminiscent of those in nematic liquid crystals (*i.e.*, systems in which a preferred axis is chosen, but the two directions along this axis are equivalent [69]) and allow A to bind to A and B to bind to B across cell-cell junctions. We assume that protein diffusion along a given edge is fast, so that the concentration of each complex is constant along edges, but keep track of slower protein exchange between edges.

Finally, the model incorporates bidirectional coupling between mechanical forces and cell polarization: On the one hand, the mechanical tensions along edges, and through them the shapes of individual cells, depend on the concentrations of the PCP proteins along those edges. On the other hand, interactions between PCP proteins favor their accumulation on shorter edges, so that cells tend to polarize along their long axes. External mechanical stresses deform cells, and thereby influence PCP alignment.

**Figure 5:**
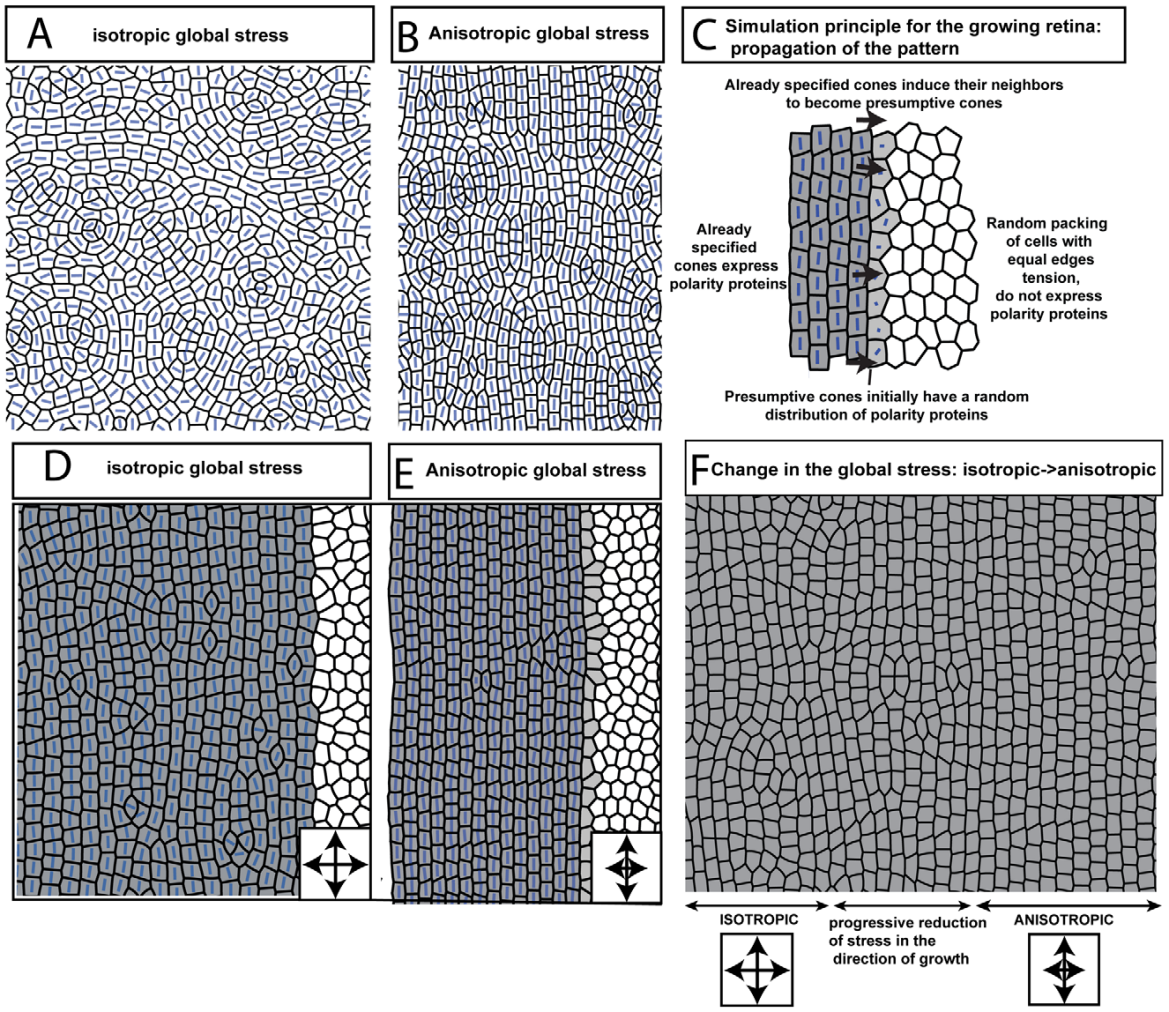
Simulation results. Blue bars indicate the axis of cell polarization from PCP; longer bars correspond to stronger polarization. **A, B**) If all cells assume the cone fate simultaneously, whether under conditions of isotopic or anisotropic global stress, a disordered packing results. **C**) In the growing retina, cone photoreceptors are generated in a propagating, linear wave. Proliferative precursor cells are shown in white and cone photoreceptors in grey. To mimic growth and differentiation at the germinal zone in the simulations, successive columns of cells are induced to assume the cone fate. **D**) Induction of cones column-by-column with isotropic mechanical stress leads to a packing that is more ordered than in panel A, but still imperfect. **E**) Induction of cones column-by-column in the presence of anisotropic mechanical stress yields straight columns. **F**) When stress anisotropy is added during a simulation, ordering improves. Double-headed arrows indicate regions of the simulated packing where the initial induction of cone fate occurred under the indicated stresses.

The precise mathematical form of the model (see below) reflects several further assumptions:

- The model allows for two types of cells, proliferative precursor cells and cone photoreceptors. We thus do not distinguish among the different cone spectral subtypes. This choice allows us to focus on behavior that arises generically from coupling PCP to cell shape and avoids the explosion of poorly constrained parameters that would result from including four different spectral fates. The model will give an accurate description of the events leading to the establishment of the cone mosaic if, during the initial stages of mosaic formation near the retinal margin, either cell-cell interactions depend weakly on spectral subtype or spectral fate has not yet been determined. While we expect that such a model will be able to capture the initial formation of cone photoreceptor columns, it may not reproduce all of the subsequent, more subtle refinements that lead to the final crystalline mosaic.
- The PCP pathway is active in cones, but both PCP proteins have concentration zero in precursors.
- We do not explicitly include Müller glia or rod photoreceptors in the model. The phenomenological parameters that govern cell shape in our model include the average effect of glial processes interposed between cone cells at the level of the OLM. On the other hand, it is unlikely that rods contribute mechanistically in an essential way to cone mosaic formation: In several teleost species with prolonged larval development, the retina contains no rod photoreceptors, but the cone mosaic is highly ordered [70]; in a zebrafish rod degeneration mutant the cone mosaic is not disrupted [71]; and in goldfish selective elimination of rod progenitors does not disrupt the cone mosaic [56].
- To model progressive growth of the mosaic at the retinal margin, at regular intervals we change all precursors that are touching a photoreceptor into a photoreceptor (Fig. 5C). Newly formed photoreceptors initially have random concentrations of polarity proteins on their edges. Instead of explicitly modeling mitosis of precursors, we allow the cone mosaic to propagate into a large field of undifferentiated precursor cells; this should be a reasonable approximation as long as dividing cells are at least a few cell diameters away from the differentiating cone photoreceptors.
- In the unperturbed adult retina, we assume the germinal zone is compressed radially (*i.e.*, perpendicular to the margin), as suggested above (“Motivation”).
- Equilibrium cell shape is determined by an effective energy (or work function; Eq. 1 below), with a quadratic area elasticity and an interfacial tension along the edges that summarizes the effects of both cell-cell adhesion and acto-myosin contractility in the adherens band. Such effective energy models have been quite successful in describing cell shape in other epithelia [37], [60]–[61], [63], [72]–[73]. In the interest of reducing the number of unknown parameters in the model, we include an interfacial energy linear in edge length, but not the additional term quadratic in cell perimeter that has been used by some authors. Although this quadratic term is necessary for precise quantitative agreement with some experiments [60], [63], it does not affect qualitative behavior in the regime relevant to this paper, in which edges are always under a significant contractile tension [67].
- PCP protein concentrations are governed by a purely phenomenological effective energy (Eq. 2 below) chosen to yield polarization and to favor high protein concentrations on shorter edges. Total protein numbers in each cell are conserved.
- Interfacial tensions vary linearly with PCP protein concentration. This dependence might reflect either direct adhesive interactions between core PCP proteins or polarized recruitment of molecules that modulate either cell-cell adhesion or the tension of the actin-myosin cortex in the adherens band.
- Vertices move in the direction of the net force exerted on them by the tensions of their adjoining edges, and edge curvatures evolve towards a balance between edge tension and the pressure difference between adjoining cells. PCP protein concentrations move downhill in the PCP effective energy (for given cell shapes and topology). We do not, however, include cross derivatives between the mechanical and PCP energies. That is, the PCP dynamics does not depend on the mechanical energy, even though the edge tensions are modulated by PCP protein concentrations, and similarly the shape dynamics is independent of the PCP energy. Thus, although it is based on two effective energies, the model is not variational in form and so is clearly non-equilibrium.
- Topological transitions in the cell packing are attempted when two vertices come within a certain cutoff distance of each other and are accepted if the net tension force on the two vertices after the transition tends to separate them further (see Methods, below).

#### Mathematical formulation

As just described, our model is based on two effective energies: a mechanical energy 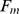 and an energy 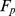 governing the dynamics of polarity proteins (compare [37]). These have the form:
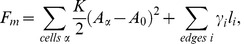
(1)where 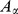 denotes the surface area of cell 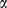 and 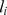 the length of edge 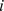, and
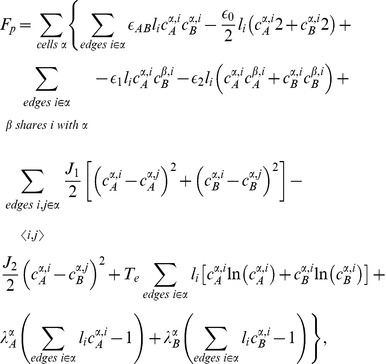
(2)where 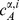 and 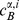 are the concentrations of polarity proteins A and B in cell 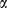 and on edge 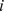; 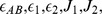 and 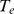 are parameters describing the interactions among PCP proteins (Fig. S5A); and 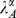 and 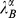 are Lagrange multipliers that keep the total number of proteins in each cell constant. The PCP energy contains quadratic interactions that favor the segregation of proteins of opposite polarities within each cell (Fig. S5A) and that explicitly depend on the side length 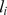 and a non-linear entropic term constraining the concentrations to be positive. An essential assumption of the model is that the tension on an edge is related to the concentration of polarity proteins on that edge according to:
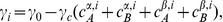
(3)where 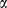 and 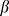 denote the two cells separated by the edge 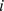.

These energies determine the system's time evolution through the kinetic equations
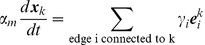
(4)

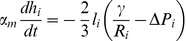
(5)

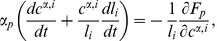
(6)where 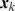 denotes the position of vertex 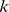, 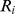 is the radius of curvature of edge 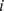, 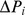 is the pressure difference between the two cells separated by edge 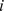, and 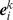 is the unit vector tangent to edge 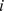 at vertex 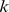 and pointing outwards from the vertex. Rather than using 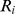, which inconveniently goes to infinity as edges become straight, to track edge curvature, we instead choose as our dynamical variable 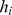, which is defined as the distance between the middle of a curved edge 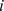 and the middle of the straight line joining the two ends of the edge. The first two equations are approximations to the gradient descent dynamics that we would obtain if we did not force edges to be circular arcs [74]: When edges can take arbitrary shapes, the functional derivative of 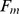 with respect to edge position is the sum of surface terms identical to the right-hand side of Eq. (4) and of local terms within a single edge proportional to 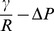, where 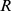 is the local radius of curvature. The right-hand side of Eq. (5) can then be viewed as a sort of average of this force over the edge, which we now demand be a circular arc; in setting this force directly equal to 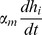, we implicitly include an 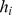-dependent mobility. Eq. (6) is derived from the assumption that the total number of polarity proteins on one edge is maintained constant, even if the length of the edge changes, in the absence of chemical reactions corresponding to a relaxation of 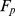.

The model is solved on a rectangular domain of size 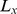 by 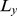 with periodic boundary conditions in both directions; to eliminate drift in the margin orientation, we fix the position of one vertical column of vertices. To introduce anisotropic global stresses, we add to the energy 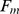 a term 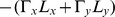 incorporating Lagrange multipliers 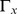 and 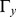 for the total size of the tissue. These multipliers correspond to external tensions and are related to imposed global external stresses 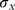 and 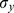 through 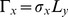 and 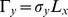. The global size of the frame is then varied according to
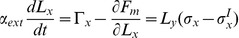
(7)

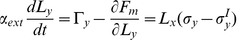
(8)where 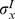 and 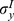 denote the (spatially averaged) internal stresses due to edge tensions and area elasticity within the cell packing. The packing was evolved quasistatically relative to the frame size variation, which imposed 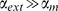. (Under Methods, below, we discuss parameter choices and robustness and describe our precise procedures for setting initial conditions and for dealing with topological transitions. Text S1 calculates the corrections that would be expected if we were to take into account the curvature of the retinal epithelium and shows that they are small for our system.)

#### Simulation results

**Figure 6:**
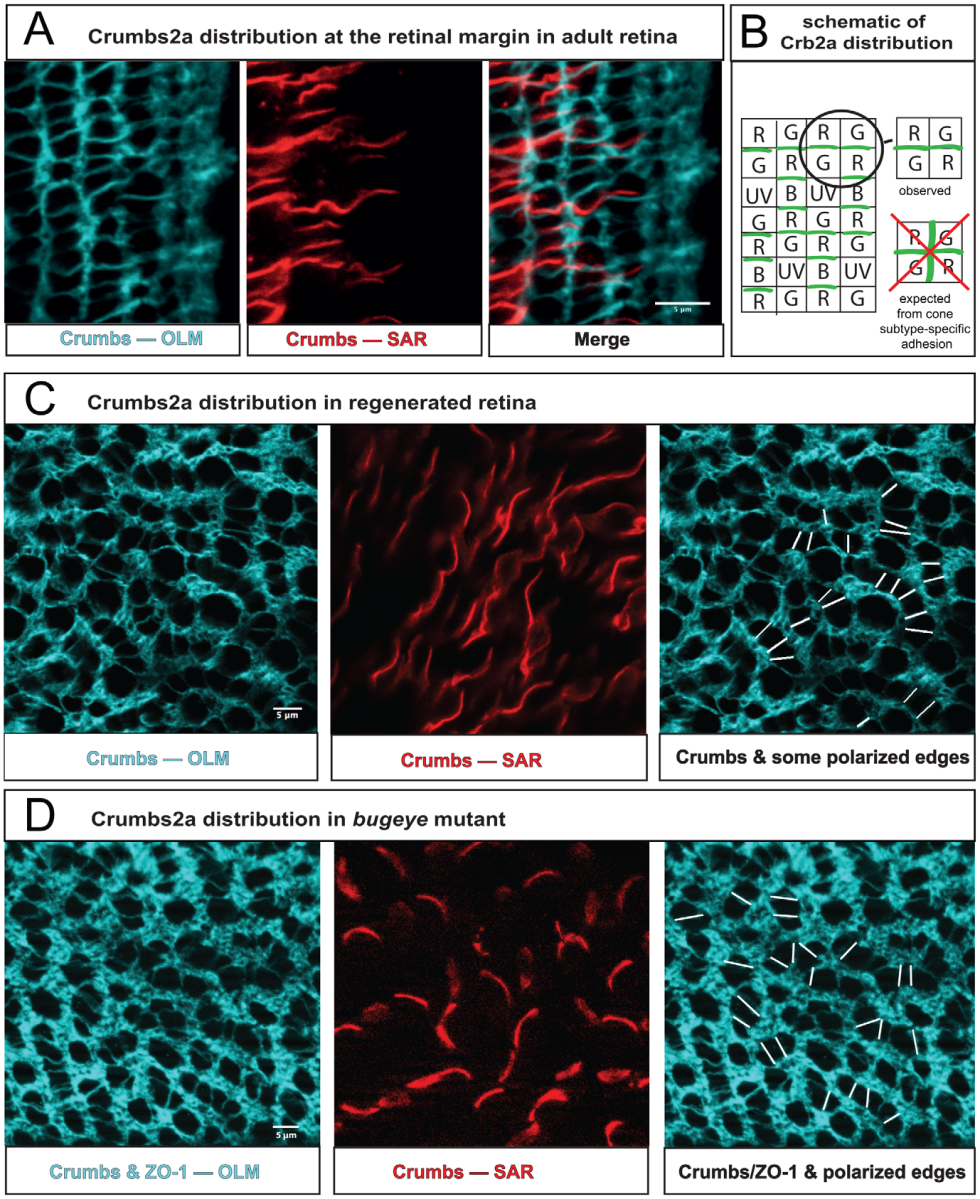
Planar cell polarity in intact and regenerated retina. **A**) Crb2a protein localized by immunocytochemistry in a flat-mount preparation at the margin of the adult retina (germinal zone to the right). The focal plane is at the level of the zonula adherens/OLM (left panel, projection of 12 confocal z slices, cyan); at the level of the inner segments/SAR of cone photoreceptors (middle panel, single confocal z slice, red); an overlay of both panels (right). See also Fig. S4J–L. **B**) Schematic illustrating Crb2a distribution along cone-cone interfaces within a column at the level of the SAR. Note that Crb2a does not localize to the orthogonal interfaces between adjacent cones across columns, as would be expected if Crb2a mediated unpolarized, but spectral-subtype-dependent, interactions between cones. **C**) At the level of the OLM, cone photoreceptors (large profiles) in regenerated retina are not organized in a rectangular lattice, but aggregate into short chains one cell wide, indicating polarized interactions (left panel). The planar polarized interfaces are verified by Crb2a localization in the SAR of these cones (middle panel). By tracing the Crb2a signal through successive focal planes in the Z-stack some planar polarized SAR interfaces between cone inner segments were associated with the corresponding cone-cone interfaces at the OLM, as indicated by white line segments (right panel). **D**) At the level of the OLM, cone profiles in the adult *bugeye* mutant are organized similarly to the regenerated retina, here visualized with a cocktail of antibodies against ZO-1 and Crb2a (left panel). Again similar to the regenerated retina, Crb2a localizes to planar polarized SAR interfaces between cone inner segments (middle panel), and some of these were traced to the cone-cone interfaces at the OLM (right panel).

We found that simulations of our model were able to replicate most of the major observed features of cone mosaic formation in zebrafish (Fig. 5 and Video S1). When the cone fate is induced in a new column of cells, their PCP axis aligns parallel to the retinal margin. With this PCP orientation, the tensions of edges perpendicular to the margin are low while those of edges parallel to it are high, leading to straight, well-ordered columns parallel to the margin, with only occasional defects (Fig. 5E). The cells in these columns have roughly rectangular or trapezoidal shapes, like those observed in adult fish retina (Figs. 4A, 6A; S1B, E, H, K). Reflecting the ordering achieved in the simulated packing, order parameters computed from the cell centroid positions were relatively high (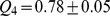 and 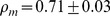, [mean 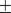
SD, 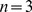]). In contrast, the values obtained for packings in the absence of both anisotropic tensions and progressive mosaic growth (Fig. 5A; compare Fig. 1E) were 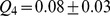 and 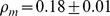 (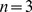), very close to the order parameters measured for embryonic retina (Fig. 1F). That the order parameter values obtained for simulated ordered packings are slightly higher than the measured order parameters of adult retinas presumably reflects the presence of sources of variability and noise in the actual biological tissue (or the fixation and imaging procedures) beyond those included in the model.

The computations also reproduce some more subtle features of our experimental observations that we did not deliberately try to include in the model. For example, in our simulations, edges parallel to the margin, between columns, often alternate between long and short, with the shorter edges, which appear slightly tilted relative to the margin, having higher PCP protein concentrations. At vertices joining long and short edges parallel to the margin, the third edge is tilted towards the shorter of the other two edges, consistent with the fact that the short edges tend to have a lower tension than the long edges. As a result, many of the edges within columns are not oriented exactly perpendicular to the columns, but are instead angled. Similar patterns are seen near the margin in adult fish (Figs. 3D, 6A; S4J).

Importantly, our model requires both progressive, column-by-column growth and anisotropic stress to produce a well-ordered cone mosaic. In the absence of either factor, errors tend to proliferate (Fig. 5B, D; Videos S2 and S3). In particular, progressive growth with isotropic stress (which we hypothesize occurs in the larval fish) yields a packing that shows significant short-ranged correlations but lacks a truly ordered lattice, just as we observed experimentally (Fig. 5D; compare Figs. 3B and 4A). Indeed, even a transient loss of stress anisotropy at the tissue scale leads to mosaic disruption. If the anisotropy is subsequently restored, the patterning process can recover, and newly created columns will be well-ordered, but the defects created while the stress was isotropic cannot readily be removed (Fig. 5F). Thus, a temporary perturbation of the retina's mechanical environment is predicted to lead to a band of disorganized cell packing within the adult retinal mosaic. The effects of removing progressive growth can be studied experimentally by, for example, ablating cone photoreceptors in the center of the retina and allowing them to regenerate (from Müller glia that function as retinal stem cells [75]–[76]). Similarly, if the intraocular pressure is altered, we expect that the stress anisotropy would be affected. We report experiments examining both of these effects in the next two sections.

We also find that the greatest regularity in cell packing generated by the model is attained when the PCP systems in neighboring cells have nematic, rather than purely polar interactions; in the strictly polar case, newly differentiating cells can choose one of two PCP orientations parallel to the margin, and columns tend to be deformed or broken at boundaries between domains with different orientations (Fig. S7 and Video S4). On the other hand, our model is robust to parameter variation, with qualitatively correct behavior persisting over at least a factor of 2 in each parameter (see Methods, below, and Fig. S6). This is hardly surprising; indeed, the arguments motivating our model indicate that it should quite generically lead to anisotropic, column-like organization of cells akin to what we observe in fish retina.

### Polarization of Crumbs2a as predicted by the mathematical model

In order to test our proposal that PCP couples with mechanical interactions to sculpt the cone mosaic, we first looked for experimental evidence of planar polarization in the retinal epithelium. The Crumbs complex–the transmembrane Crumbs2a (Crb2a) protein and associated intracellular scaffolding proteins in the MPP5 (membrane palmitolated protein 5) family (*Drosophila* ortholog *stardust* and zebrafish orthologs *Nok* and *Ponli*)–localizes to the subapical region (SAR) in the inner segment region of zebrafish cone photoreceptors [77]–[79]. This complex is important in maintaining apical-basal polarity and the integrity of the adherens junctions at the OLM and is thought to mediate cell-cell adhesion both between photoreceptors and Müller glia [50] and between photoreceptors [78].

As cone photoreceptors differentiate, for example in the larval retina from 4 to 10 dpf, the inner segment elongates, as does the SAR, as delimited by Crb2a immunostaining (Fig. S4A–C). In the adult, the SAR interface between inner segments of red-green double cones extends up to 50 µm apical to the OLM (Fig. S4D–I). In contrast, thin lamellar processes of Müller glia surround each cone profile at the level of the OLM, as identified by ZO-1 immunostaining (Figs. S1J–L, S4D), but in the adult retina, these processes extend at most ∼15 µm apically beyond the OLM (Fig. S4G–I). Above the Müller glial processes, the inner segments (SAR) of cones have the opportunity for direct cell-cell contacts without intervening glia (Fig. S4E–H). The inner segments/SAR of the red-green double cone pairs, in particular, are tightly apposed and exclude all Müller glial processes from the level of the OLM apically (Fig. S4I).

We found that the distribution of Crb2a protein in cone photoreceptors is co-localized with ZO-1 at the level of the zonula adherens in the OLM, but shows planar polarization at the level of the inner segments within the SAR. In this region of direct cell-cell contacts between cones, the Crb2a protein exhibits a polarized distribution aligned with the rectangular cone lattice, as predicted by the mathematical model, with Crb2a enriched along interfaces between adjacent cones within columns compared with interfaces between columns (Fig. 6A, S4J–L). High levels of Crb2a in the SAR are thus correlated with relatively weak interfacial tensions while low levels indicate higher tensions. Importantly, this planar polarization is observed near the retinal margin, before significant numbers of rods that might prevent contacts between cones in different columns have inserted. Indeed, it is interesting to note that rods subsequently insert along interfaces with low Crb2a concentrations, consistent with reports that knockdown of Crumbs favors insertion of transplanted rods into murine retina [80].

### Disrupted mosaics as predicted by the mathematical model

One of the motivations for introducing PCP, and hence planar polarized mechanical interactions between cone photoreceptors, into our model was the observed linear fragments of cone columns even in the embryonic or larval retina or remnants (Figs. 1E, G, 4A), where long-ranged order is lacking. The model predicts that similar ordered domains should be observed wherever the cone mosaic is not fully crystalline. To verify this prediction, we examined cone photoreceptor cell packing in regenerated retinal tissue in adult zebrafish. If photoreceptors in a region of the neural retina in adult zebrafish are ablated through exposure to very intense light, the Müller glia in the affected region re-enter the cell cycle and form scattered clusters of neurogenic progenitor cells that regenerate a new complement of cone photoreceptors [75]–[76]. The resulting restored photoreceptor mosaic, however, lacks the crystalline order seen in the undamaged adult retina [27]. Instead, we find that the regenerated retina contains short, curvilinear chains of cones, a single cell wide, with intervening spaces filled with rods (Fig. 6C). The organization of cones in the regenerated retina is closer to simulations of packing of cones being specified simultaneously under a global isotropic stress, where disconnected regions of rectangular order form in all directions (Fig. 5A). It also somewhat resembles that of the larval remnant in the adult retina (Fig. 1G), though the larval remnant has far fewer rods. This striking morphology is strong evidence of anisotropic, polarized interactions between individual cones, independent of any crystalline ordering. Moreover, in the SAR of the regenerated cones, Crb2a localizes preferentially to interfaces within the curvilinear chains of cones (Fig. 6C), suggesting that these groups may be viewed as fragments of cone cell columns organized by PCP-dependent junctional structures, just as the mathematical model predicts.

Our model also suggests that perturbations of the mechanical environment of the retina as cones are differentiating should lead to defects in the cone mosaic. Consistent with this prediction, we find that the cone mosaic is disturbed in *bugeye* mutant fish (Fig. 6D; compare simulations Fig. 5D, F). The *bugeye* locus encodes the Low density lipoprotein receptor-related protein 2 (Lrp2), a large transmembrane receptor with multiple identified ligands [81]. In the eye, Lrp2 is expressed in the ciliary epithelium of the anterior segment of the eye and in the retinal pigment epithelium behind the neural retina, but not in the neural retina itself. The phenotype of the *bugeye* mutants in adult fish includes enlarged eyes, elevated intraocular pressure (IOP), and thinner epithelial layers with decreased photoreceptor density (consistent with mechanical stretching induced by the IOP), but with variable penetrance; the severity and time of onset of the defects varies significantly from one animal to the next and even between the two eyes of a single fish [81]. We detected cone mosaic disruption only in enlarged eyes of *bugeye* fish (Fig. 6D). When imperfections were observed, they were strongly reminiscent of the defects found in the regenerated regions (Fig. 6C) and in simulations of our model in the absence of a global stress anisotropy (Fig. 5D). Because Lrp2 is not expressed in photoreceptor neurons, the effects of the mutation must be transmitted to the photoreceptor layer either through a secondary signal from the retinal pigmented or ciliary epithelium or through changes in mechanical properties like IOP. (One effect of such a secondary signal might be to affect cell proliferation or death, but no increase in apoptosis is observed in the photoreceptor layer, and, although there is conflicting evidence as to whether and how proliferation is affected, experiments to date do not suggest dramatic enough changes in proliferation to induce the near-complete loss of long-ranged order of Fig. 6D [81]–[82]). Although we cannot rule out the possibility of some unknown signal from the non-neural ocular epithelia that is permissive for crystalline mosaics, we favor the latter hypothesis: The loss of long-ranged crystalline order in a mutant with elevated IOP suggests that large changes in the IOP may disrupt the pattern of tissue-scale mechanical stresses necessary for global alignment of the mosaic pattern, as predicted by our mathematical model.

## Discussion

### The geometry of photoreceptor packing in the retinal epithelium

In the crystalline cone mosaic of the adult zebrafish retina, both the spectral fates of cone photoreceptor cells and their shape and packing in the apical plane of the retina exhibit precise patterns. Here, we have focused on the planar packing of cones, which appears to arise roughly simultaneously with the determination of cone cell fate and the differentiation of the SAR as represented by elaboration of the cone inner segment. To gain insights into the mechanisms producing the rectangular lattice packing, we first characterized the arrangement of cone and rod photoreceptors in mature zebrafish retina, which we showed is dominated by circumferential columns of closely apposed cones, a single cell wide, oriented parallel to the site of cone genesis in the germinal zone at the peripheral retinal margin. The cones in a column thus represent a cohort of cells of approximately the same age. Cones in successive columns are also aligned in radiating rows, and rod photoreceptors are mostly constrained to the interstices between cone columns. This rectangular lattice pattern contrasts with the more disordered cone cell packing in the embryonic and larval retina, which largely lacks rods and has reduced numbers of red and green cones [15], but which nevertheless shows evidence of short, linear chains of cones, reminiscent of the columns in the adult cone mosaic, albeit without long ranged order. We also examined the emergence of epithelial order as new cones are generated at the retinal germinal zone and contrasted this with the relative lack of order in two experimental conditions: 1) when cones in light-damaged, adult zebrafish regenerated in central retina from unaligned, scattered neurogenic foci, and 2) when cones were generated in the *bugeye* mutant zebrafish, which has altered intraocular mechanics.

Cone photoreceptor genesis in both larval and adult zebrafish occurs only in the germinal zone at the circumference of the retina, and in both cases the retina grows by adding successive annuli of cells at the perimeter. Nonetheless, a truly ordered cone mosaic is only seen in adults, not in larval fish. Thus, progressive, spatially restricted differentiation cannot, by itself, explain the appearance of a crystalline cell packing. Instead, the emergence of regular cone packing coincides with the maturation of the ciliary epithelium in the anterior segment at the end of larval development, with the concomitant production of aqueous humor and the resultant intraocular pressure, and with the simultaneous formation of the annular ligament, a mechanical constraint overlying the retinal germinal zone in the anterior segment [28]. We therefore hypothesize that the tissue-scale mechanical environment may play a central role in regulating local cell shapes and packing in the retinal epithelium as the cone mosaic is formed and that the development of the anterior segment may lead to a significant change in this mechanical environment. Building on this idea and on our experimental observations, we propose that circumferential columns of cones form through a feedback between mechanical tension at cell-cell interfaces and PCP, with an anisotropic mechanical stress, possibly imposed by the annular ligament, providing an overall orientational signal. A mathematical model incorporating both cell mechanics and PCP reveals that such a global mechanical stress, together with progressive growth and addition of cells at the retinal margin, is sufficient to robustly assemble the new cones into a coherent rectangular lattice.

The model is supported by our observations of a polarized distribution of Crumbs2a protein in differentiating cones near the retinal margin, as well as in mature cones in the patterned areas of adult fish. The Crumbs transmembrane proteins define the apical membrane of epithelial cells, including photoreceptors in *Drosophila* and vertebrates, and are implicated in cell-cell adhesion through a poorly understood mechanism [50], [77], [83]–[84]. The intracellular domain of Crumbs proteins is associated with a macromolecular complex of scaffolding proteins, including the MPP5 proteins such as zebrafish Nok [83]. Genetic evidence suggests that the Crumbs/Nok apical junctional complex mediates photoreceptor-photoreceptor adhesion in zebrafish: In a zebrafish mutant with nonfunctional N-cadherin, the adherens junctions of the OLM are destabilized and the apicobasal polarity of the retinal epithelium is destroyed, but the photoreceptors nevertheless develop an intrinsic apical-basal polarity and self-associate into small, scattered, spherical “rosettes” with apical surfaces pointing toward the center; in the absence of functional Nok protein, rosettes fail to form [83]. Further, Nok mutant photoreceptors fail to localize Crumbs protein to the SAR of the inner segments, and photoreceptor cells with mutant Nok protein transplanted into a wild type retina show increased mobility (consistent with reduced cell-cell adhesion) when viewed by time-lapse microscopy in a living zebrafish embryo [83]. Even more relevant to the present results are recent reports on the subcellular localization of two proteins, a novel Nok/MPP5 family member in zebrafish, Ponli (Photoreceptor-layer-nok-like), and Crumbs2b, both of which are expressed exclusively in red, green, and blue cones and which show polarized localization to the SAR coincident with the localization demonstrated here for Crb2a, *i.e.* at the interfaces between cones within a column, but not between columns [79], [84].

In further support of our model, we have here reported fragments of cone cell columns in cones regenerating within the adult retina at a distance from the germinal zone and the annular ligament [75]–[76] and in the *bugeye* mutant, in which the ocular globe enlarges dramatically as a consequence of increased intraocular pressure [81]; these column fragments exhibit a planar-polarized Crb2a distribution that corresponds very closely to that seen in the cone cell columns of unperturbed retina. Very recently, Zou *et al.* have observed similar column fragments in transgenic fish with secreted Crb2b extracellular fragment [84].

Additional evidence for strong adhesive interactions between cone photoreceptors comes from morphological observations. The double cones are of special interest: These tightly apposed pairs of cones are found in many vertebrate taxa, though not in placental mammals [85]. Electron microscopy reveals specialized, subsurface, membranous cisternae, located ∼90 Å beneath and parallel to the apposing plasma membranes of the inner segments in the double cone pairs [54], [86], *i.e.* in the region where the Crumbs complex is localized, and electron dense material has been observed in the extracellular space between these apposing membranes [87]. Finally, double cones dissociated from the retina remain physically attached to each other at the interface of their inner segments [88]–[89].

### A mathematical model of PCP and cell shape

The defining characteristic of the model presented here is the feedback loop encompassing mechanical stresses, cell shape, and PCP: Anisotropic stresses, whether externally imposed on the epithelial sheet or generated internally, tend to deform cells, and the resulting elongated cell shapes in turn favor cell polarization along a particular direction in the epithelial plane. Finally, PCP leads to anisotropic tensions along cell-cell interfaces, closing the loop. Importantly, cells in our model will spontaneously polarize even in the absence of tissue-scale anisotropic stresses imposed by the annular ligament, which serve only to encourage all cells to align in the same direction (and hence to line up in columns); the model's behavior is thus insensitive to the exact magnitude of the anisotropy. In the presence of a global stress anisotropy, these interactions are sufficient to reproduce the observed packing of cone photoreceptors near the retinal margin, including not only the presence of aligned columns, but also such unexpected features as the tendency of cell-cell interfaces within columns to tilt relative to the average column direction and the rotation of the average direction of the cone cells' long axis as they leave the germinal zone. Consistent with our hypothesis of a central role for PCP in determining cell packing, we have moreover observed planar-polarized protein distributions in adult cone mosaics and a tendency of cone photoreceptors in regenerated retina to form chains, suggestive of planar-polarized interactions between these cells.

Our model makes several further predictions that we have not yet been able to test experimentally. Most obviously, we expect that the retinal epithelium supports anisotropic mechanical stresses. On the scale of the entire tissue, this should take the form of different diagonal stress tensor components in the radial and circumferential directions. At the level of individual cells, cell-cell interfaces are expected to have disparate tensions depending on their orientation relative to the direction of the cell's planar polarization. In fact, it should be possible to correlate interfacial tensions with the concentrations of proteins (including perhaps Crb) implicated in PCP. In principle, tension anisotropy on both scales can be measured with appropriate forms of laser microsurgery combined with live cell imaging [60], [64]. In addition, it might be possible to infer the tensions along individual interfaces from a careful analysis of cell shapes and, in particular, of the angles at which interfaces meet at vertices [90]. More broadly, our analysis suggests that changes to the neural retina's overall mechanical environment should disturb the precision of the cone mosaic. Thus, mutations or experimental treatments affecting the eye anterior segment—and especially the annular ligament—might be expected to disrupt the cone mosaic; for example, if it were possible to ablate the annular ligament entirely, we would predict that the crystalline cone mosaic would revert to something closer to the disordered packing seen in the larval retina. Similar effects might also occur as a result of sufficiently large and sustained changes to the intraocular pressure, though in this case global mechanical effects might be difficult to separate from the consequences of physiological stress responses within individual photoreceptor neurons.

### Outlook

One striking feature of the cell packing at the retinal margin in the adult is the sudden shift from a haphazard cell array in the germinal zone to the orderly columns characteristic of mature retina. This is to be contrasted with the more gradual rearrangements seen in another prominent example of a crystalline cell packing resulting from front-like growth: In the *Drosophila* eye imaginal disc, a rough lattice of isolated cells fated to become R8 photoreceptors is first selected from within an essentially disordered cell packing. These R8s then signal to surrounding cells to induce successive waves of differentiation, with significant changes in cell shape and position in most cases coming more gradually and only after fate specification [91]–[92]. The abrupt appearance of columns in fish retina suggests a much tighter integration of spectral fate specification and morphogenesis.

Also of note is our observation of planar polarized Crb protein localization in cells near the retinal margin. The Crumbs complex, which includes transmembrane Crumbs proteins and associated intracellular proteins MPP5 and Patj, is thought to work in concert with the Par-3 complex, which includes the cytoplasmic proteins Par-3 (Bazooka in *Drosophila*), Par-6, aPKC, and Cdc42, to define distinct apical membrane domains in epithelial cells [50], [93]–[95]. More recently, studies in *Drosophila* have implicated many of these same proteins—including, in one case, Crb [96]—in PCP, especially in systems where PCP is intimately linked with polarized contraction of cell-cell interfaces [48], [94]–[95], [97]–[101]. Our results suggest a related role for Crb in fish retina Significantly, in the fly systems, apical proteins have higher concentration on edges with lower tensions, and we likewise observe preferential Crb localization to (presumptively) lower tension edges in fish. Our observations thus hint that much of the machinery that establishes apico-basal polarity may be reused to regulate planar polarized cell movements in vertebrates, just as it is in flies.

It is well established that PCP can lead to anisotropic tensions along cell-cell interfaces, and thereby to cell shape changes or even to large-scale tissue remodeling. This is perhaps most dramatically seen in convergent extension [48], but the PCP pathway is also required in other processes, ranging from oriented cell division to the establishment of the hexagonal cell packing in *Drosophila* wing imaginal discs [13], [49] or the morphogenesis of Kupffer's vesicle in zebrafish [102]. On the other hand, recent evidence also indicates that both static cell packing defects and the dynamics of cell division and changes in packing topology can influence PCP in wing discs [37]–[38]. Mathematical models have been able to reproduce and explain many of these observations [37]–[38], [48]–[49]. This prior work, however, focuses largely on unidirectional influences either of PCP on cell mechanics or of cell mechanics on PCP. By including interactions in both directions, we are able to paint a more complete portrait of morphogenetic processes involving PCP. Indeed, the studies just cited show that such feedback loops must exist in wing discs. Likewise, columns of cells reminiscent of those seen in mature fish retina appear in several other systems where PCP is known to be active, most notably in the ventral epidermis of *Drosophila* embryos, where a similar interplay among PCP, polarized tensions, and cell shape changes may well be at work [96], [99]–[100]. In convergent extension, it remains unclear how the global PCP orientation is faithfully maintained in the face of large-scale cell movements; as these rearrangements are themselves dependent on PCP, this question can only be addressed with models that integrate the dynamics of PCP and of cell motion. Our model is thus likely to find applications in investigations of many other systems.

## Methods

### Experimental methods

#### Ethics statement

All procedures were approved by the University Committee on Use and Care of Animals at the University of Michigan.

#### Zebrafish lines

Transgenic lines expressing enhanced green fluorescent protein (EGFP) in rods, *Tg(-3.7rho:EGFP)kj2* [53] or in UV cones, *Tg(-5.5opn1sw1:EGFP)kj9* [21] were a gift from Dr. Shoji Kawamura. In the transgenic line *Tg(gfap:EGFP)mi2002*, EGFP is driven by regulatory elements of the zebrafish glial fibrillary acidic protein and in the retina is expressed selectively in Müller glia [103]. The double transgenic line, *mi2009*, expressing EGFP in UV cones and mCherry in blue cones, was generated by crossing the transgenic line *Tg(-5.5opn1sw1:EGFP)kj9* with the transgenic line *Tg(-3.2opn1sw2:mCherry)mi2007*. The transgenic line *Tg(-3.2gnat2:EGFP)ucd1* [52] expresses EGFP in all cones under the control of the cone transducin alpha promoter and was a gift from Dr. Susan Brockerhoff.

The *mi2007* line was generated with the miniTol system [104] as follows. The *mCherry* coding sequence was dropped out of *pmCherryN1* (Clontech, Mountain View, CA) with *BamHI* and *NotI* and ligated into the *BamHI/NotI* site of the *-3.2opn1sw2:EGFP* plasmid (a gift from Shoji Kawamura; [21]) to generate the construct *-3.2opn1sw2:mCherry*. The *-3.2opn1sw2:mCherry* construct was cut with DraIII and blunt ended, the *-3.2opn1sw2:mCherry* cassette was then dropped out with *BglII* and ligated into the *EcoRV* and *BglII* site of the *pMiniTol2* vector (a gift from Dr. Steve Ekker). The *pMiniTol2 -3.2opn1sw2:mCherry* construct was then co-injected with transposase mRNA into one-cell stage zebrafish embryos. Germline founders were identified and one founder was used to generate the subsequent generations of *Tg(-3.2opn1sw2:mCherry)mi2007*. Fish were raised and maintained using standard protocols [105] in E3 media and aquaria water at 28.5°C.

#### Histology

Two different dissection methods were used to prepare retinal flat mounts from adult zebrafish to visualize the cellular organization at the apical retinal surface:

In the first method, which is the standard retinal flat-mount, the retina is isolated from the other ocular tissues, and mounted flat on a microscope slide. Adult zebrafish, 2–4 months post-fertilization (mpf) were anesthetized in Tricane (Sigma). With Vannas micro-scissors, a circumferential cut was made at the limbus and the entire anterior segment (ciliary epithelium, iris epithelium, lens and cornea) was removed. A radial cut was made along the ventral axis of the eye cup for orientation, the optic nerve was severed, and the retina was flushed out of the eyecup onto a piece of Parafilm by applying a stream of phosphate buffered saline. A large drop of fixative (4% paraformaldehyde in 0.1 M phosphate buffer, pH 7.4 with 5% sucrose) was placed on the tissue and short relaxing cuts were made along the retinal perimeter. After 15 minutes a second piece of Parafilm was placed on top of the drop of fixative and a 2.5 g weight was placed on top to flatten the retina. The tissue was placed in a humid chamber fixed flat for 30 minutes, then removed from the Parafilm sandwich and immersion-fixed for another 45 minutes. After fixation the tissue was rinsed in 0.1M phosphate buffer with 5% sucrose, 3 times for 20 minutes each and mounted on a microscope slide with the photoreceptor side down. The photoreceptor layer was imaged by focusing down through the retina from the vitreal surface.

A second method was designed to visualize the germinal zone and the new cone photoreceptors generated at the peripheral retinal margin, which is at the limbus of the eye, the junction between sclera and cornea. This region of the retina is often damaged in the dissection method above. Adult zebrafish, 2–4 mpf were anesthetized, the eyes were enucleated and placed cornea side down on a piece of Parafilm. A hole was made with a microscalpel in the back of the eye at the optic nerve head and radial cuts were made with Vannas microscissors along the dorsal/nasal and ventral/temporal axes through the sclera, choroid, pigmented epithelium, and neural retina. The four flaps were gently laid open and the lens removed. The tissue was transferred to a drop of fixative and processed as described above except a 15 g weight was used to flatten the eyecup. After fixation the tissue was rinsed in 0.1M phosphate buffer with 5% sucrose, 3 times for 20 minutes each and mounted on a microscope slide with the scleral side down.

For whole mount preparations of larval zebrafish eyes, embryos were treated with PTU (1-phenyl-2-thiourea) at 12 hours post-fertilization to block melanin formation in the retinal pigmented epithelium [105]. The larval fish were fixed at 4–5 days post-fertilization in 4% paraformaldehyde in 0.1 M phosphate buffer, pH 7.4 with 5% sucrose for 2 hours at room temperature and rinsed three times for 20 minutes each. Prior to whole mount immunocytochemistry (see below) the eyes were dissected out of the head of the larval fish; processed eyes were mounted under a coverslip on a microscope slide.

Retinal cryosections 6 µm thick were prepared as previously published [106].

#### Immunocytochemistry

Whole mount immunohistochemistry was carried out immediately after post-fixation rinses. Isolated retinas, flattened whole eyes from adults or whole intact eyes from larvae, were blocked for 1 hour at room temperature with 10% normal goat serum, 1% Tween, 1% Triton X-100, 1% DMSO in phosphate buffered saline with 0.1% sodium azide. Primary antibodies were diluted with 2% normal goat serum, 1% Tween, 1% Triton X-100, 1% DMSO in phosphate buffered saline with 0.1% sodium azide, and the tissue was incubated overnight at room temperature. The following antibodies and dilutions were used: mouse anti-Zonula Occudins-1, generated against a human recombinant ZO-1 peptide, amino acids 334–634, (Invitrogen Corporation, Camarillo, CA), 1∶200; zs4 monoclonal antibody (Zebrafish International Resource Center, Eugene, OR), 1∶10, which recognizes the extracellular domain of Crb2a [77]. After overnight incubation the tissue was washed three times, 20 minutes each in phosphate buffered saline with 1% Tween, 1% Triton X-100, and 1% DMSO, then incubated with anti-mouse DyLight 649 or DyLight 549, 1∶200, (Jackson ImmunoResearch Inc., West Grove, PA) overnight at room temperature and washed as above. Following the final wash the tissue was mounted on a microscope slide and coverslipped under ProLong Gold (Invitrogen Corporation, Camarillo, CA). Immunocytochemistry on cryosections was performed as published [106].

Images were collected on a Zeiss Axio Image ZI Epifluorescent Microscope (Carl Zeiss Microimaging Inc., Thornwood, NY) or a Leica SP5 Scanning Confocal Microscope (Leica Microsystems, Bannockburn, IL 60015), or an Olympus FluoView 500 Laser Scanning Confocal microscope (Olympus America, Inc., Melville, NY).

### Image processing

Adobe Photoshop CS5 Extended (Adobe Systems Inc., San Jose, CA) and ImageJ 1.43u (http://rsb.info.nih.gov/ij) were used for post-acquisition processing of digital images. Any digital adjustments to contrast, gain, color, filtering, and layer properties were applied to the entire image. Some figures include maximum projections created from selected layers in confocal Z-stacks. In some cases when overlaying multiple fluorescent channels in a Z-stack projection, selected regions of an individual layer were masked to avoid parallax.

The order parameters extracted from experimental images were obtained by segmenting the cell profiles at the level of the OLM with a watershed algorithm. To avoid including Müller cell or rod profiles in the analysis, small cells with area below a cut-off were removed from the segmentation by morphological shrinking. In the margin area, we also removed regions that appeared morphologically distorted; we have verified that those regions are filled with Müller cell processes (Fig. S1J–L). These regions were detected by enforcing a minimal threshold for the ratio between the region area and the area of the convex envelope of the region. From the segmented image, the positions of cell centroids were computed and used to determine order parameters (Figs. S2 and S3).

### Orientational order parameter 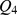


An orientational order parameter reflecting the four-fold rotational symmetry of a perfect cone mosaic was used to evaluate quantitatively the closeness of an arrangement of cells to a rectangular periodic lattice. Inside each cell, a cross is specified (Fig. 2A, blue line) whose orientation reflects the neighbors position. To do so, for every orientation of the cross the plane is separated into four quadrants (Fig. 2A, dotted line) and the distance between the arms of the cells and the closest neighbor within the quadrant to which the arm belongs is calculated. The cross orientation is then set by minimizing the sum of these distances. This allows assigning a cross for every cell in the epithelium characterizing the packing geometry (Fig. 2B). An average order parameter for the cross orientations can then be obtained for a group of cells by computing two components, 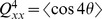 and 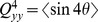 where 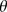 refers to the angle with the horizontal of one of the cross arms. This specific functional form is required by the π/2 rotational invariance of the crosses. The magnitude of the order parameter is then given by 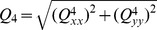 and satisfies 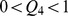. The value of 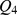 reflects the level of ordering: 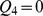 corresponds to completely random orientation, whereas 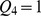 reflects a perfect alignment of the crosses (Fig. 2C). Cells assembled on a perfect rectangular lattice would have an order parameter 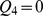.

### Mathematical model

Our model's basic biological content and mathematical formulation is discussed under Results above. Here, we elaborate on some of the finer technical points.

#### Topological transitions

T1 transitions [66] were incorporated into the simulations by defining a cut-off length 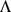 below which an edge is shrunk to a single vertex. In this process, the proteins found on the small edge were equally redistributed to the neighboring edges within each cell, according to the following rules, where the subscripts 1 to 4 refer to neighboring edges, the variables without subscripts to the edges that are shrunk to zero, 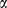 and 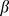 to the two cells separated by the edge being shrunk, and 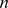 and 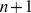 to the times before and after the topological transition (see Fig. S5B for a schematic): 
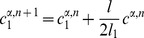
, 
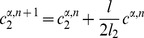
, 
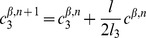
 and 
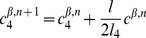
.

4-fold vertices were subsequently tested for topological transition and creation of a new straight edge of length slightly larger than 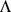 connecting two 3-fold vertices. A transition was considered favorable when the force acting on the newly formed edge favored an increase of its length, *i.e.* when
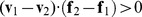
where 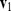 and 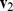 denote the positions of the two vertices connected by the newly formed edge, and 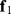 and 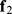 are the total forces exerted on each of the two vertices due to the tensions of its connected edges (Fig. S5B). If the two possible resulting topologies were both allowed by this criterion, the topology with the largest positive scalar product was chosen.

Different choices can be made on how much polarity protein the newly formed edge receives. We chose to redistribute polarity proteins according to the following rules, where the new edge is formed between the vertices connecting respectively the edges 1 and 3 and 2 and 4 (Fig. S5B): 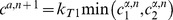, 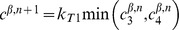, 
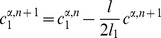
, 
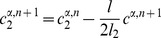
, 
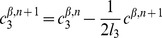
 and 
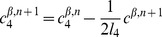
. These rules ensure that a certain amount of protein is redistributed to the newly formed edge while conserving the total number of proteins in each cell. For 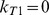, there are no polarity proteins on the newly formed edge, which in our framework leads to a new edge with a high tension and therefore promotes 4-fold vertices. For 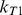 of order 1, formation of new edges is favored, which can later lead to the disappearance of the new edge following redistribution of the polarity proteins and hence to cycles of formation and disappearance of an edge. In the simulations presented in this paper we chose 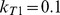.

#### Initial conditions

Simulations are performed by starting from Voronoi tessellations of squares of side *L* on which *N* points are randomly distributed. The resulting cell packing is subsequently relaxed to equilibrium assuming uniform tensions along the edges, so that the edges meet at 120° at vertices. When cells are subsequently assigned a cone cell fate, they receive a random distribution of polarity proteins on their edges. Each concentration is chosen randomly between 0 and 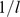 where, 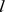 is the edge length, with the constraint that the sum of the concentrations of each kind of protein within one cell is equal to 1. To initiate simulations of growing, propagating patterns, a first column of cone cells is established by assigning a cone cell fate to all cells crossed by a vertical line within the packing. Subsequent cone cells are then propagated by induction from this initial column, as described above.

#### Parameters

The parameters of our model can be normalized through the definition of a reference tension 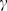, a reference length 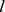 equal to the side length of a cell in a honeycomb packing filling the square of side 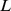 (and thus satisfying 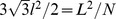, where 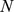 is the total number of cells), reference interaction strength between proteins 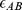, and two reference timescales, a mechanical timescale 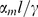 and a polarity protein timescale 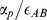 All other parameters can be normalized to these quantities. We set 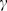 as the tension of precursor cells, leaving the two quantities 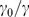 and 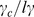 as free parameters for cone cells. In the limit where each polarity protein accumulates on one edge within the cell and assuming the cell polarities are correlated across the tissue, typical tensions then lie between 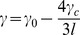 for edges with high polarity protein concentrations and 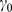 for edges that have no PCP proteins (Fig. S5C).

In simulations involving only cone cells (Fig. 5A–B) the following set of parameters was used: 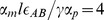, 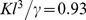, 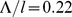, 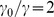, 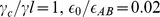, 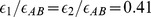, 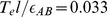, 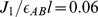, 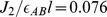. Parameters describing the PCP dynamics were empirically chosen to generate aligned polarity in a stretched honeycomb packing: The simulations were performed by relaxing a honeycomb packing with initial uniform edge tension and random concentrations of polarity proteins, taking periodic boundary conditions on a frame of fixed size. To simulate the effect of anisotropic stress (Fig. 5), an initial isotropic packing produced by relaxation of a Voronoi tessellation was compressed quasistatically along one direction until it reached 2/3 of its initial size, before starting the simulation with polarity proteins.

Simulations with propagating fronts of differentiation (Fig. 5D–E) also include a second type of cells corresponding to cone progenitors in the germinal zone. These cells were chosen to have no polarity proteins on their edges and a uniform tension 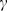. Other parameters were identical to those for cone cells. During propagation, the frame size was relaxed with the choice 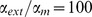.

Simulations of a propagating pattern with varying external global stress (Fig. 5F) were done with an additional rule: the area elastic modulus of cone cells was increased to 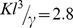 while keeping the former value of 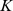 for progenitors. This led to less deformation of the already assembled retina following a modification in the global stress to establish anisotropic stress in the packing. We also verified that this rule did not affect our results for propagation under isotropic and anisotropic condition, only leading to retina with larger cones.

#### Robustness

To study the robustness of our results to parameter variation, we performed simulations of a propagating pattern with an anisotropic applied stress, as described, while varying each model parameter in succession. We found that variations between 60 and 200% of 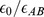, 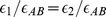, 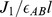, 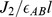, and 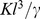 did not qualitatively affect the patterns produced. We also verified that for relative velocities 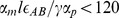 the patterns produced were still ordered.

We more systematically investigated the effect of varying 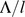, 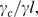 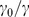 and 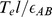 on the regularity of the pattern produced (Fig. S6). The pattern generation mechanism was likewise robust to variation of these parameters. Away from the robust region, topological defects consisting of line dislocations could be observed, similar to the defects observed in non-propagating packings. Interestingly, the pattern formation requires a large enough cut-off edge length 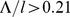 to ensure a sufficient regularity of the unspecified packing to be patterned. Similarly, a small enough value of 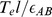 was necessary to ensure that polarity proteins where strongly concentrated on two opposite edges. The model was relatively robust to 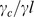 and to the related anisotropy of edges tensions within each cell, with generation of regular packings for 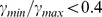. As expected, decreasing the value of 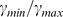 led to packings with straighter interfaces between columns.

#### Numerical methods

An implicit-explicit scheme with an adaptive step size method was used to simulate the dynamics of the system defined by Eqs. (4) through (6) (see Text S1 for details).

## Supporting Information

Figure S1 **Identification of cell profiles at the apical surface of the adult zebrafish retina.** A–C) Retinal flat-mount from a young adult transgenic zebrafish (*ucd1*) in which the cone transducin alpha promoter drives expression of the EGFP reporter. A, C) All cones are green. B, C) cell boundaries at the level of the OLM are labeled with ZO-1 (white). B) UV cones (magenta stars) can be recognized by their large apical profiles relative to the other cones and rods. Red-green double cone pairs within a vertical column are tightly apposed with flattened interfaces (white arrows in B and also in E and H). D–F) Retinal flat-mount from a young adult transgenic zebrafish (*kj2*) in which the rod opsin promoter drives expression of the EGFP reporter. D, F) All rods are green. E, F) Cell boundaries at the level of the OLM are labeled with ZO-1 (white). F) The initial rods insert between vertical cone columns at the intersections between red, green, blue and UV cones in adjacent columns. G–I) Retinal flat-mount from an older adult rod transgenic zebrafish (*kj2*). Rods continue to accumulate between adjacent vertical cone columns. J–L) Flat-mount at the margin of the retina from a young adult transgenic zebrafish (*mi2002*) in which the promoter from the glial-specific gene, *gfap*, drives expression of the EGFP reporter in Müller glial cells. The germinal zone is at the right. J, L) Müller glia cells at the apical surface (green); thin lamellar processes completely surround all rod and cone photoreceptors at the OLM, and Müller glia also account for the polygonal profiles in the germinal zone and adjacent region where cones are differentiating (magenta arrows in K and L; also see Fig. 3A).
(TIF)

Figure S2 **Example of the segmentation procedure.** A) Original image, B) Segmentation result, C) Overlay (original image has been darkened and segmentation lines thickened for visualization purposes).
(TIF)

Figure S3
**Fourier transform of cell centroid positions.** Top row: examples of Fourier transform for points distributed randomly, periodic columns with random relative shifts, and points distributed on a square lattice. Middle row: simulation results for different values of the parameter 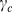. Lower row: experimental data.(TIF)

Figure S4 **Differentiation of cone photoreceptors and elaboration of the apical process.** A) Retinal cryosection from larval transgenic (*mi2009*) fish at 4 days post-fertilization (dpf), in which blue and UV cones express the reporters mCherry and EGFP (pseudocolored blue and magenta, respectively), which fill the cytoplasm of the cells. The OLM (arrow) is labeled by ZO-1 immunostaining (yellow). The developing inner and outer segments of the cones project apically beyond the OLM. B) Larval *mi2009* fish at 4 dpf immunolabeled for Crb2a (yellow). Note that the Crb2a protein extends beyond the OLM (arrow) on the plasma membrane of the inner segments. C) By 10 dpf, the cone inner and outer segments have elongated further and the Crb2a protein also extends further apically on the inner segments. The arrow indicates the level of the OLM. D) Differential interference contrast (DIC) image of a retinal cryosection from an adult zebrafish, immunolabeled with ZO-1 (yellow) to label the OLM (arrow). The inner and outer segments of cones extend apically; the UV cones are the shortest, the blue (B) cones are longer, and the red and green (RG) double cones are the longest. E) A DIC image with immunolocalization of Crb2a protein on the inner segments of the cones. The black arrow indicates the OLM. The inset shows the Crb2a protein at the interface between red and green double cones (white arrows). F) Same image as panel E without the DIC channel. G) Differential interference contrast (DIC) image of a retinal cryosection from an adult transgenic zebrafish (*mi2002*), expressing a fluorescent reporter in Müller glia (cyan), and immunolabeled with Crb2a (yellow). H) Same image as panel E without the DIC channel. The processes of Müller glia (white arrows) extend apically beyond the OLM, but not as far as the Crb2a (yellow). Müller processes do not separate the interface between the inner segments of red and green double cone pairs, which have strong staining for Crb2a (yellow arrows). I) Left half: DIC image of a retinal cryosection from an adult transgenic zebrafish (*mi2002*), expressing a fluorescent reporter in Müller glia (cyan), and immunostained with *zpr1*, which labels red and green cones (magenta). Right half: same image without the DIC channel. The processes of the Müller glia (white arrow) are not interposed between the inner segments of red and green double cone pairs (magenta arrow). J–L) Individual optical sections from a z-stack confocal image of Crb2a immunolabeling near the retinal margin in a retinal flat-mount (cropped version from the same image series shown in Figure 6A). Panel J is at the level of the OLM, panel K is the subapical region (SAR) at 2.5 µm from the OLM, and panel L is the SAR at 5 µm from the OLM. Identity of cone photoreceptor subtypes is indicated by asterisks: red, green, blue, and UV (magenta), respectively. In the SAR, Crb2a has a planar polarized distribution – it is expressed at higher levels at the interfaces of red, green, and blue cones within a column. The retinal surface is curved, so the left and right sides of each panel are more apical than the center (and thus show a somewhat more polarized Crb2a distribution in J and K).
(TIF)

Figure S5
**Model components.** A) Schematic of quadratic interactions in the polarity protein effective energy 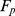. Only interactions involving the concentration 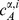 are represented. B) Redistribution of polarity proteins in topological transitions. Left: shrinking of an edge to a 4-fold vertex; the polarity proteins on the vanishing edge are redistributed onto neighboring edges. Right: formation of a new edge from a 4-fold vertex; a minimal amount of polarity proteins is redistributed onto the newly formed edge. C) Schematic illustrating how cells adopt a rectangular shape in the simulations. Left: with uniform tension, cells typically adopt hexagonal shapes where edges meet at vertices with 120 degree angles. Right: when the polarity protein dynamics is turned on, polarity proteins accumulate on opposite edges of cells, which results in an decrease of edge tension on 2 opposite sides and an increase in tension on the other 4 sides. To satisfy force balance, the cells must then deform their shapes towards a nearly rectangular shape. When minimum and maximum tensions in a cell satisfy 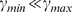, the cell shape is close to a rectangle and, in a perfectly regular packing, the length of the edges with polarity proteins is approximately 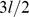, where 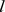 is the length of a side in the original hexagonal packing. Assuming each polarity protein is concentrated on one of two opposite edges in the cell, the concentration reached is about 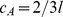, 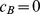 or 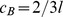, 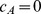. Counting the polarity proteins on both sides of the edges, this results in the estimates of 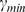 and 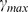 indicated below the schematic.(TIF)

Figure S6
**Robustness analysis of the formation of a regular packing in our simulations.** Columns, from left to right: nematic order parameter from the distribution of PCP proteins; solid order parameter obtained from the Fourier transform of the cell centroid positions; 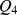 order parameter; examples of the pattern produced for extreme values of the parameters. The red line in the middle column indicates a rough empirical criterion (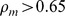) for the order parameter values above which our simulations show an ordered rectangular packing without defects resembling the experimental pattern. Black points correspond to parameters for simulations presented in the text. Values were obtained from 3 simulations with 500 cells for each point; error bars = SEM.(TIF)

Figure S7
**Model behavior with a polar PCP interaction.** In this model variant, PCP parameters are chosen to favor apposition of A and B proteins across interfaces over A and A or B and B. (Parameters 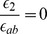, 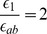, 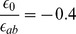, 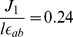, 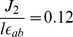, 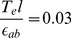, other parameters as described in the text). The simulation was started with one line of cells with a uniform polarity. Following this first line, each new row of cells is specified with a random distribution of polarity protein, and no global polarity cue is given. A) Example of simulation results: the cell packing does not show rectangular order. B) Snapshots of simulations illustrating the mechanism leading to patterning defects: polarity can point up or down, leading to domain formation. Because the resulting configuration is not favorable, the polarity reorients when the next column is specified, leading to polarity misalignment and eventually to a defect in cell packing.(TIF)

Figure S8
**Mapping from a hemispherical to a planar surface.** For a hemisphere of radius 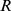, Cartesian coordinates 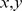 in the plane are related to the polar and azimuthal angles 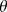 and 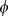 on the hemisphere as 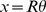 and 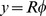.(EPS)

Text S1 **Supplementary theoretical and computational methods.**
(PDF)

Video S1 **Simulation of the progressive growth of a cone photoreceptor mosaic similar to that observed near the retinal margin in adult, wildtype fish; the cone fate is induced and the PCP pathway is activated in successive columns of cells at regular intervals, as described in the text.**
(MOV)

Video S2 **Simulation of the relaxation of a cell packing with polarity proteins influencing edge tensions, under a global isotropic stress.** The PCP pathway is activated in all cells at the start of the movie.
(MOV)

Video S3 **Simulation of the relaxation of a cell packing with polarity proteins influencing edge tensions, under a global anisotropic stress (the packing is compressed along the horizontal direction).**
(MOV)

Video S4 **Simulation of the progressive growth of a retinal cone mosaic, but with polar rather than nematic interactions between polarity proteins.**
(MOV)
