# Supplementary figures and images for: Coupling Mechanical Deformations and Planar Cell Polarity to Create Regular Patterns in the Zebrafish Retina
Source: PLoS Comput Biol. 2012 Aug 23;8(8):e1002618. doi: 10.1371/journal.pcbi.1002618 (PMC3426565; doi:10.1371/journal.pcbi.1002618)

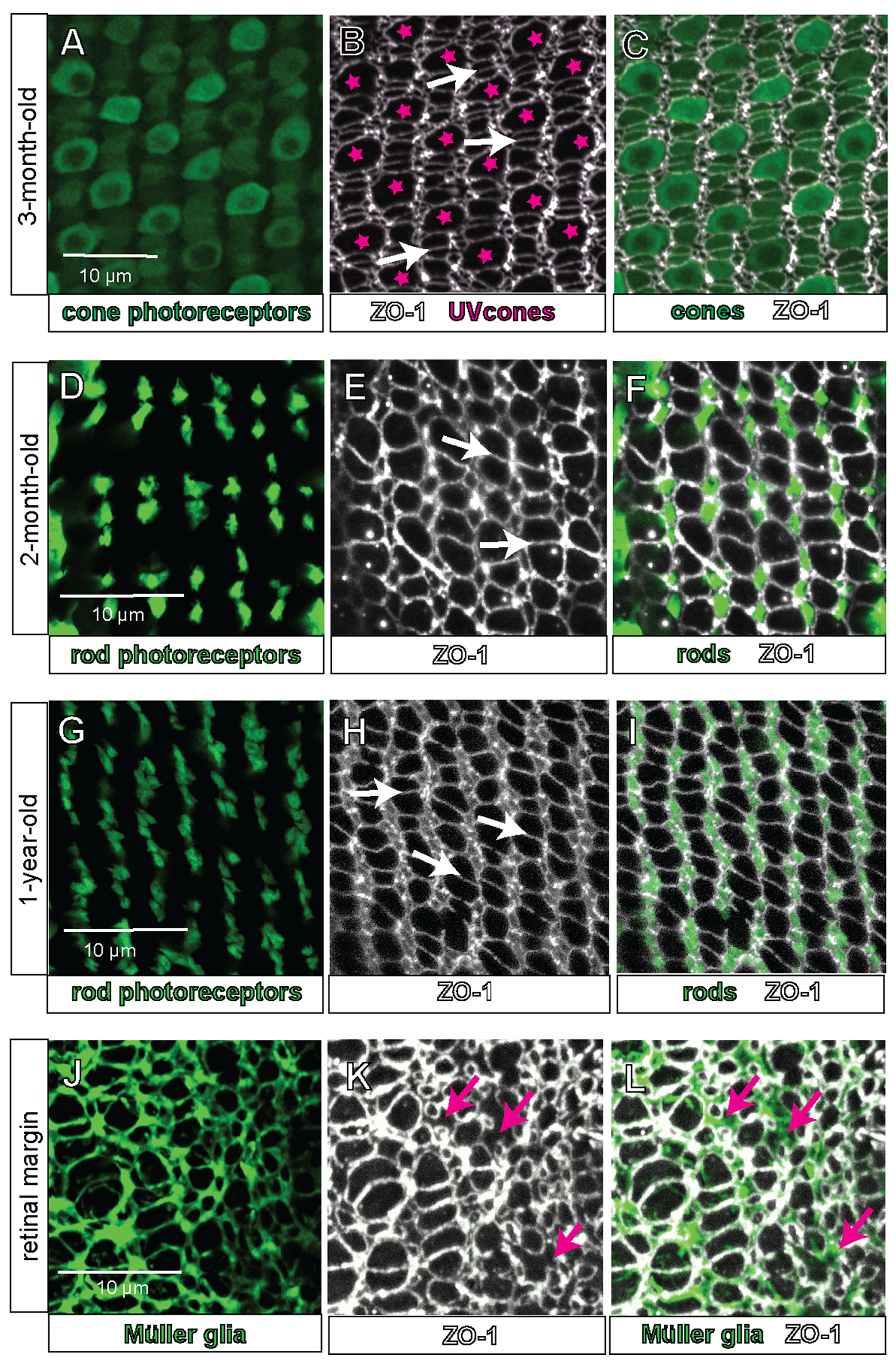

Supplement: Figure S1 — Identification of cell profiles at the apical surface of the adult zebrafish retina. A–C) Retinal flat-mount from a young adult transgenic zebrafish (ucd1) in which the cone transducin alpha promoter drives expression of the EGFP reporter. A, C) All cones are green. B, C) cell boundaries at the level of the OLM are labeled with ZO-1 (white). B) UV cones (magenta stars) can be recognized by their large apical profiles relative to the other cones and rods. Red-green double cone pairs within a vertical column are tightly apposed with flattened interfaces (white arrows in B and also in E and H). D–F) Retinal flat-mount from a young adult transgenic zebrafish (kj2) in which the rod opsin promoter drives expression of the EGFP reporter. D, F) All rods are green. E, F) Cell boundaries at the level of the OLM are labeled with ZO-1 (white). F) The initial rods insert between vertical cone columns at the intersections between red, green, blue and UV cones in adjacent columns. G–I) Retinal flat-mount from an older adult rod transgenic zebrafish (kj2). Rods continue to accumulate between adjacent vertical cone columns. J–L) Flat-mount at the margin of the retina from a young adult transgenic zebrafish (mi2002) in which the promoter from the glial-specific gene, gfap, drives expression of the EGFP reporter in Müller glial cells. The germinal zone is at the right. J, L) Müller glia cells at the apical surface (green); thin lamellar processes completely surround all rod and cone photoreceptors at the OLM, and Müller glia also account for the polygonal profiles in the germinal zone and adjacent region where cones are differentiating (magenta arrows in K and L; also see Fig. 3A). (TIF) [file pcbi.1002618.s001.tif]

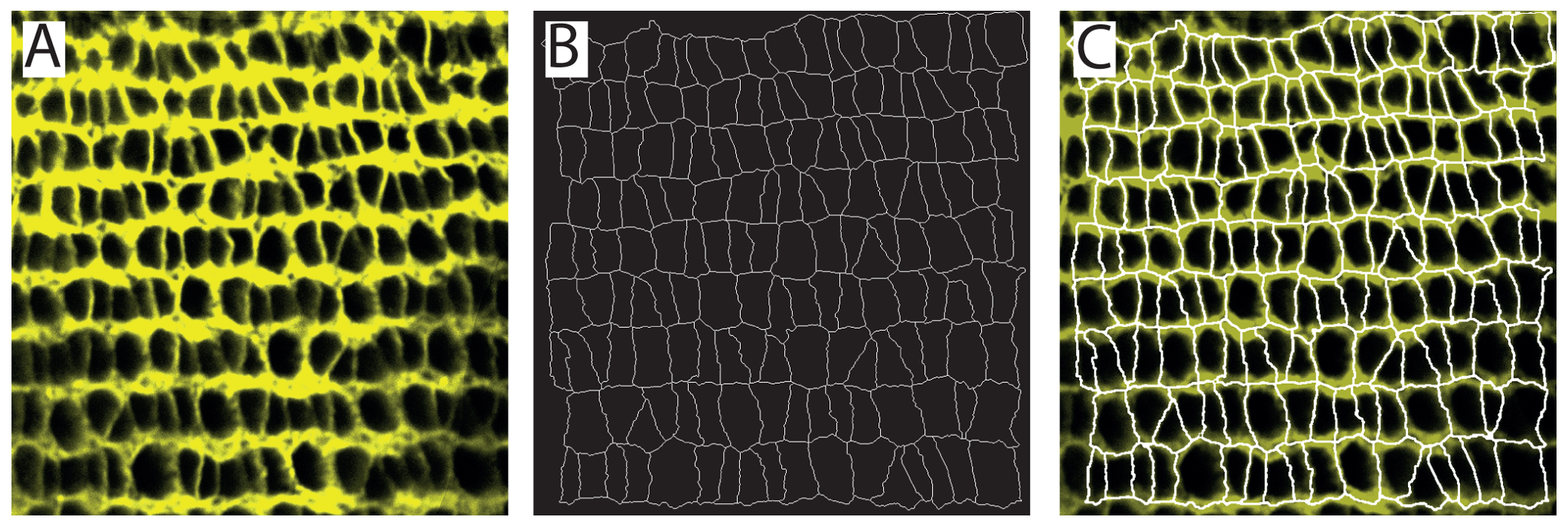

Supplement: Figure S2 — Example of the segmentation procedure. A) Original image, B) Segmentation result, C) Overlay (original image has been darkened and segmentation lines thickened for visualization purposes). (TIF) [file pcbi.1002618.s002.tif]

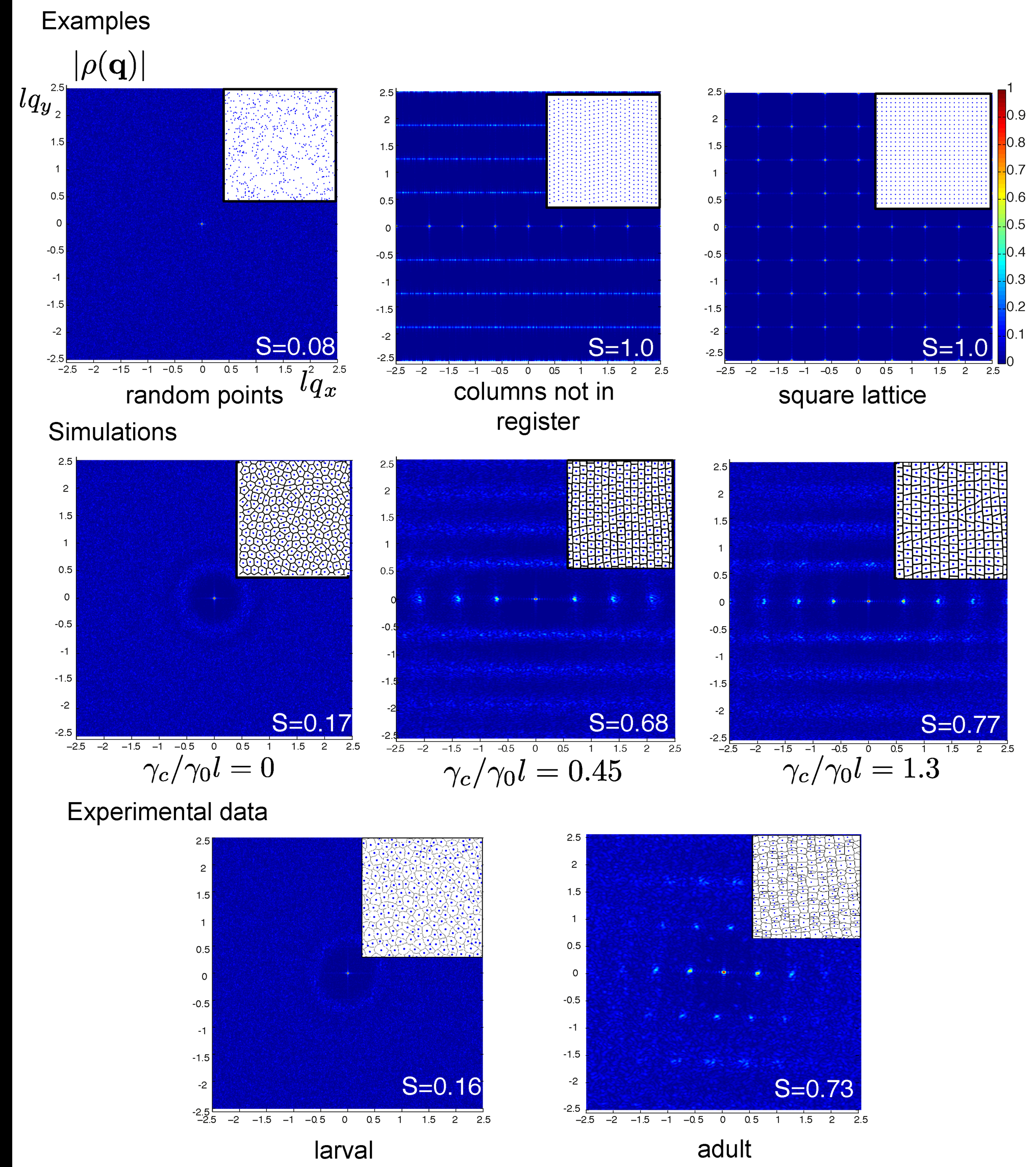

Supplement: Figure S3 — Fourier transform of cell centroid positions. Top row: examples of Fourier transform for points distributed randomly, periodic columns with random relative shifts, and points distributed on a square lattice. Middle row: simulation results for different values of the parameter . Lower row: experimental data. (TIF) [file pcbi.1002618.s003.tif]

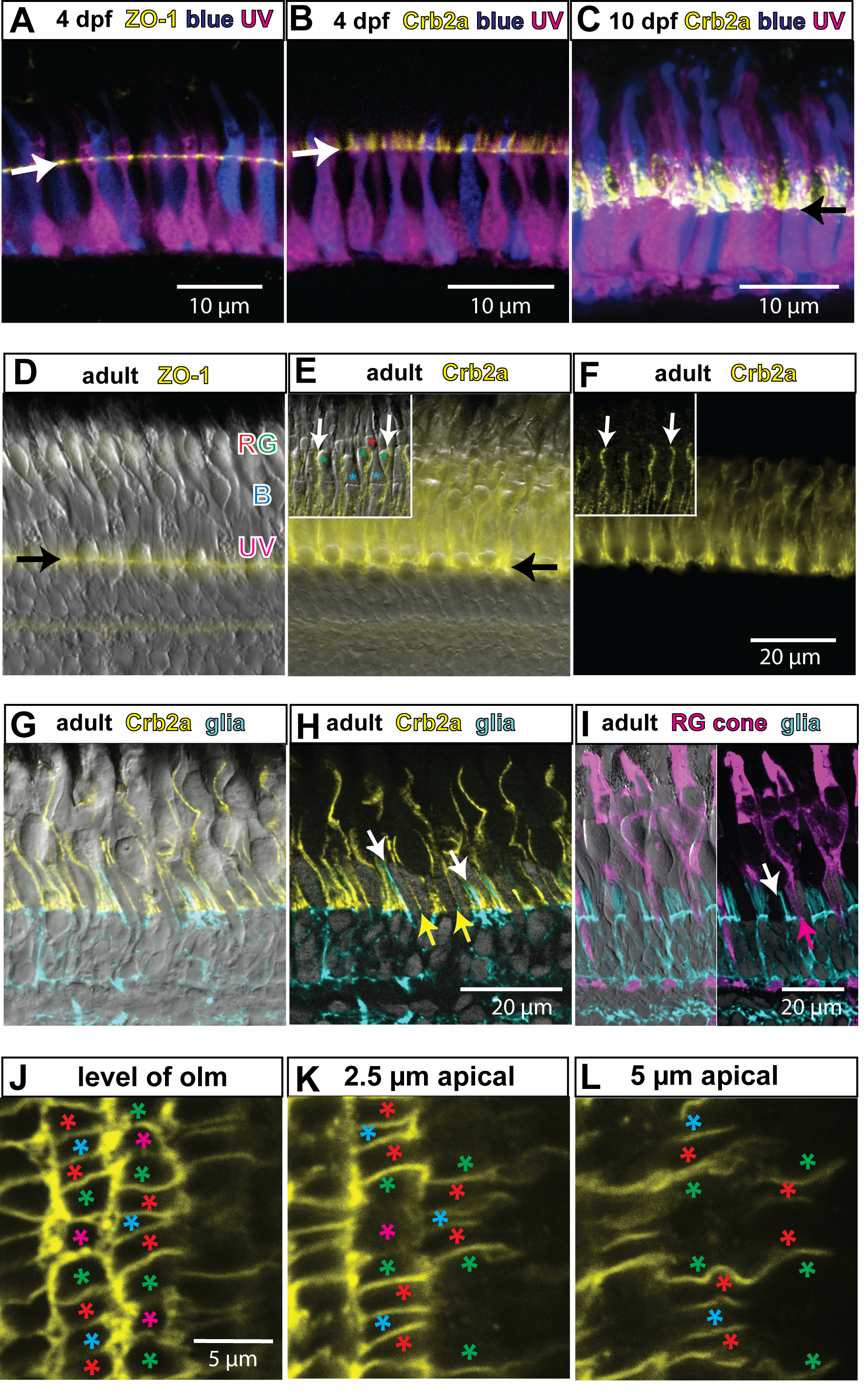

Supplement: Figure S4 — Differentiation of cone photoreceptors and elaboration of the apical process. A) Retinal cryosection from larval transgenic (mi2009) fish at 4 days post-fertilization (dpf), in which blue and UV cones express the reporters mCherry and EGFP (pseudocolored blue and magenta, respectively), which fill the cytoplasm of the cells. The OLM (arrow) is labeled by ZO-1 immunostaining (yellow). The developing inner and outer segments of the cones project apically beyond the OLM. B) Larval mi2009 fish at 4 dpf immunolabeled for Crb2a (yellow). Note that the Crb2a protein extends beyond the OLM (arrow) on the plasma membrane of the inner segments. C) By 10 dpf, the cone inner and outer segments have elongated further and the Crb2a protein also extends further apically on the inner segments. The arrow indicates the level of the OLM. D) Differential interference contrast (DIC) image of a retinal cryosection from an adult zebrafish, immunolabeled with ZO-1 (yellow) to label the OLM (arrow). The inner and outer segments of cones extend apically; the UV cones are the shortest, the blue (B) cones are longer, and the red and green (RG) double cones are the longest. E) A DIC image with immunolocalization of Crb2a protein on the inner segments of the cones. The black arrow indicates the OLM. The inset shows the Crb2a protein at the interface between red and green double cones (white arrows). F) Same image as panel E without the DIC channel. G) Differential interference contrast (DIC) image of a retinal cryosection from an adult transgenic zebrafish (mi2002), expressing a fluorescent reporter in Müller glia (cyan), and immunolabeled with Crb2a (yellow). H) Same image as panel E without the DIC channel. The processes of Müller glia (white arrows) extend apically beyond the OLM, but not as far as the Crb2a (yellow). Müller processes do not separate the interface between the inner segments of red and green double cone pairs, which have strong staining for Crb2a (yellow arrows). I) Left half: [file pcbi.1002618.s004.tif]

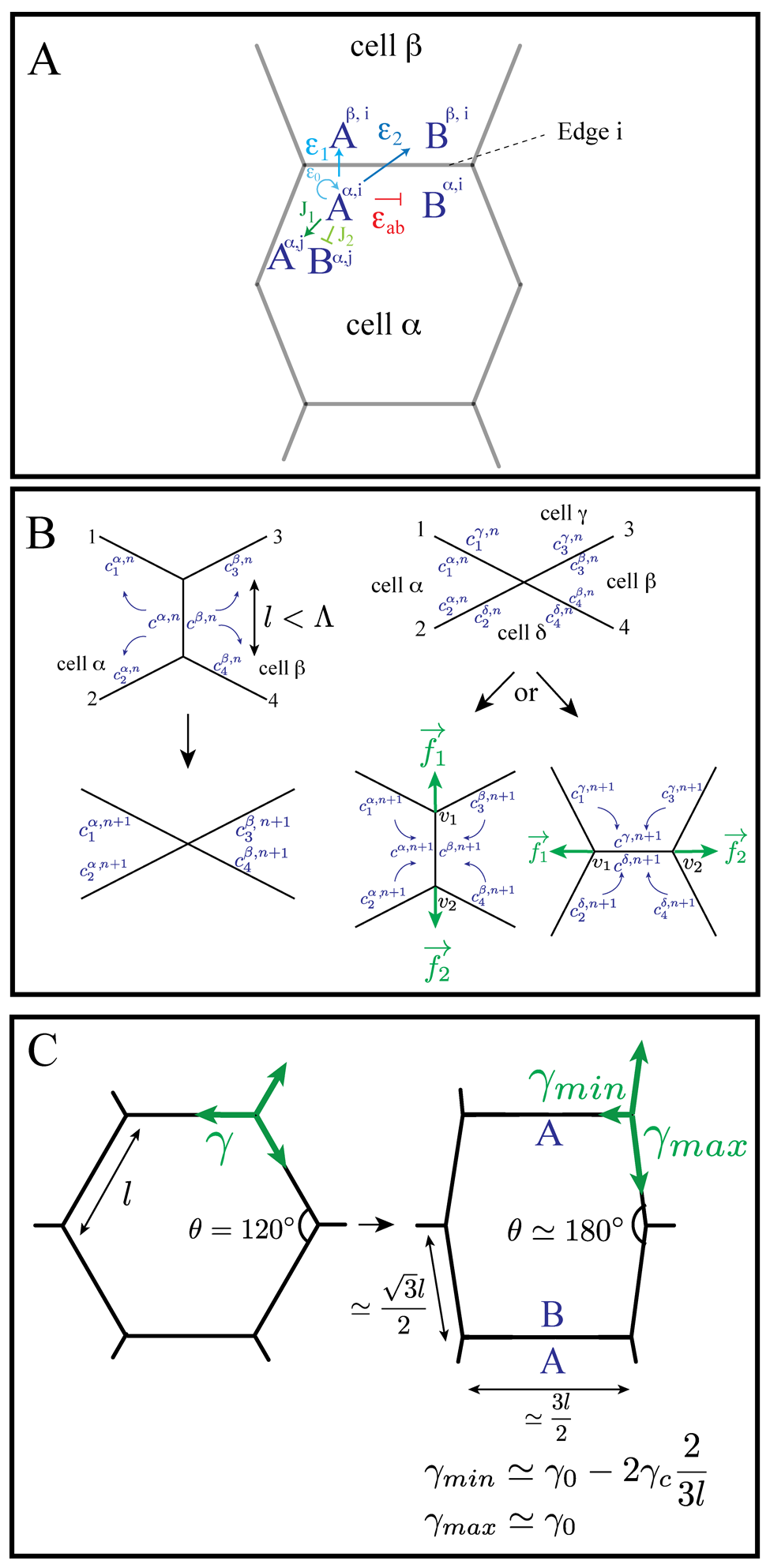

Supplement: Figure S5 — Model components. A) Schematic of quadratic interactions in the polarity protein effective energy . Only interactions involving the concentration are represented. B) Redistribution of polarity proteins in topological transitions. Left: shrinking of an edge to a 4-fold vertex; the polarity proteins on the vanishing edge are redistributed onto neighboring edges. Right: formation of a new edge from a 4-fold vertex; a minimal amount of polarity proteins is redistributed onto the newly formed edge. C) Schematic illustrating how cells adopt a rectangular shape in the simulations. Left: with uniform tension, cells typically adopt hexagonal shapes where edges meet at vertices with 120 degree angles. Right: when the polarity protein dynamics is turned on, polarity proteins accumulate on opposite edges of cells, which results in an decrease of edge tension on 2 opposite sides and an increase in tension on the other 4 sides. To satisfy force balance, the cells must then deform their shapes towards a nearly rectangular shape. When minimum and maximum tensions in a cell satisfy , the cell shape is close to a rectangle and, in a perfectly regular packing, the length of the edges with polarity proteins is approximately , where is the length of a side in the original hexagonal packing. Assuming each polarity protein is concentrated on one of two opposite edges in the cell, the concentration reached is about , or , . Counting the polarity proteins on both sides of the edges, this results in the estimates of and indicated below the schematic. (TIF) [file pcbi.1002618.s005.tif]

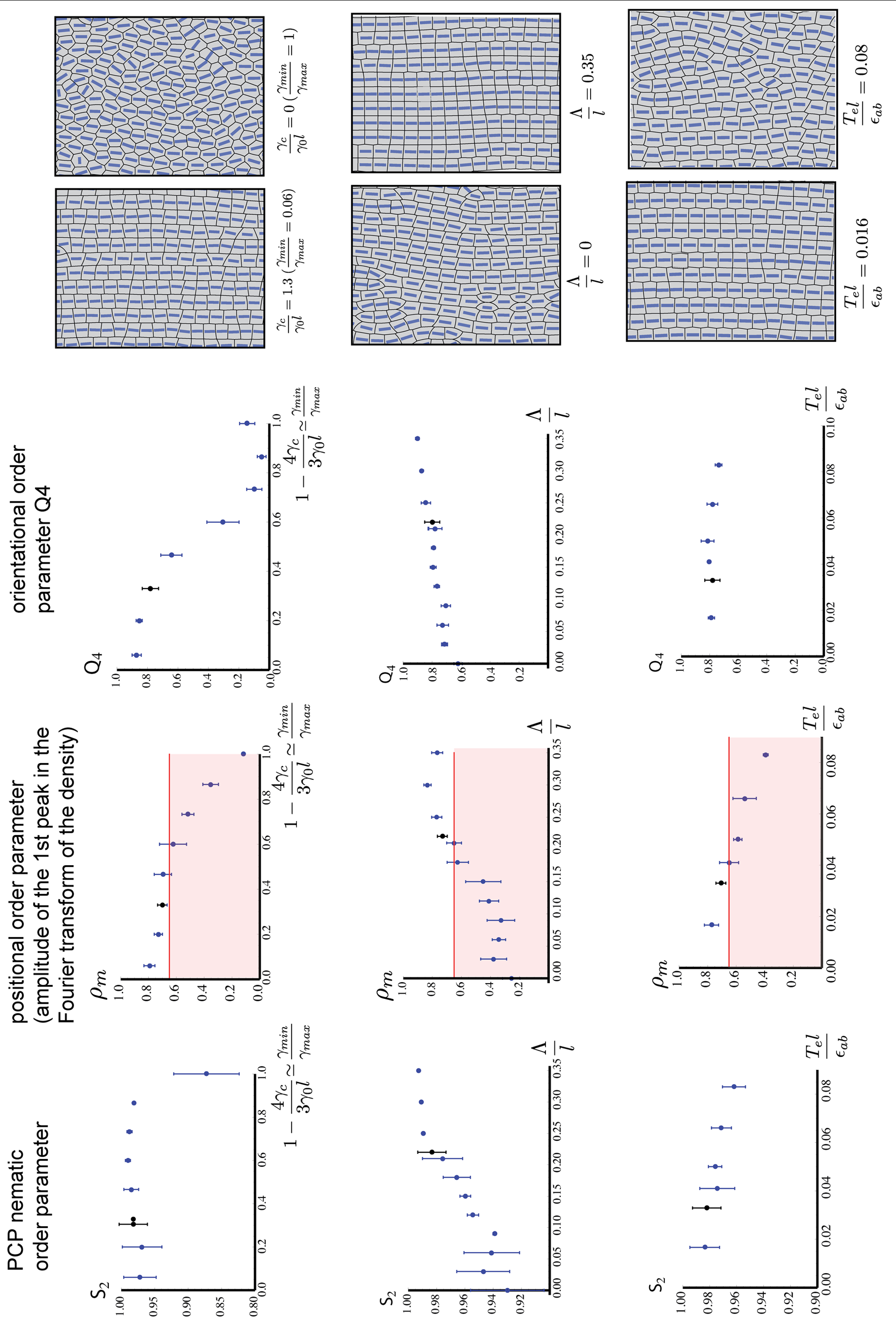

Supplement: Figure S6 — Robustness analysis of the formation of a regular packing in our simulations. Columns, from left to right: nematic order parameter from the distribution of PCP proteins; solid order parameter obtained from the Fourier transform of the cell centroid positions; order parameter; examples of the pattern produced for extreme values of the parameters. The red line in the middle column indicates a rough empirical criterion () for the order parameter values above which our simulations show an ordered rectangular packing without defects resembling the experimental pattern. Black points correspond to parameters for simulations presented in the text. Values were obtained from 3 simulations with 500 cells for each point; error bars = SEM. (TIF) [file pcbi.1002618.s006.tif]

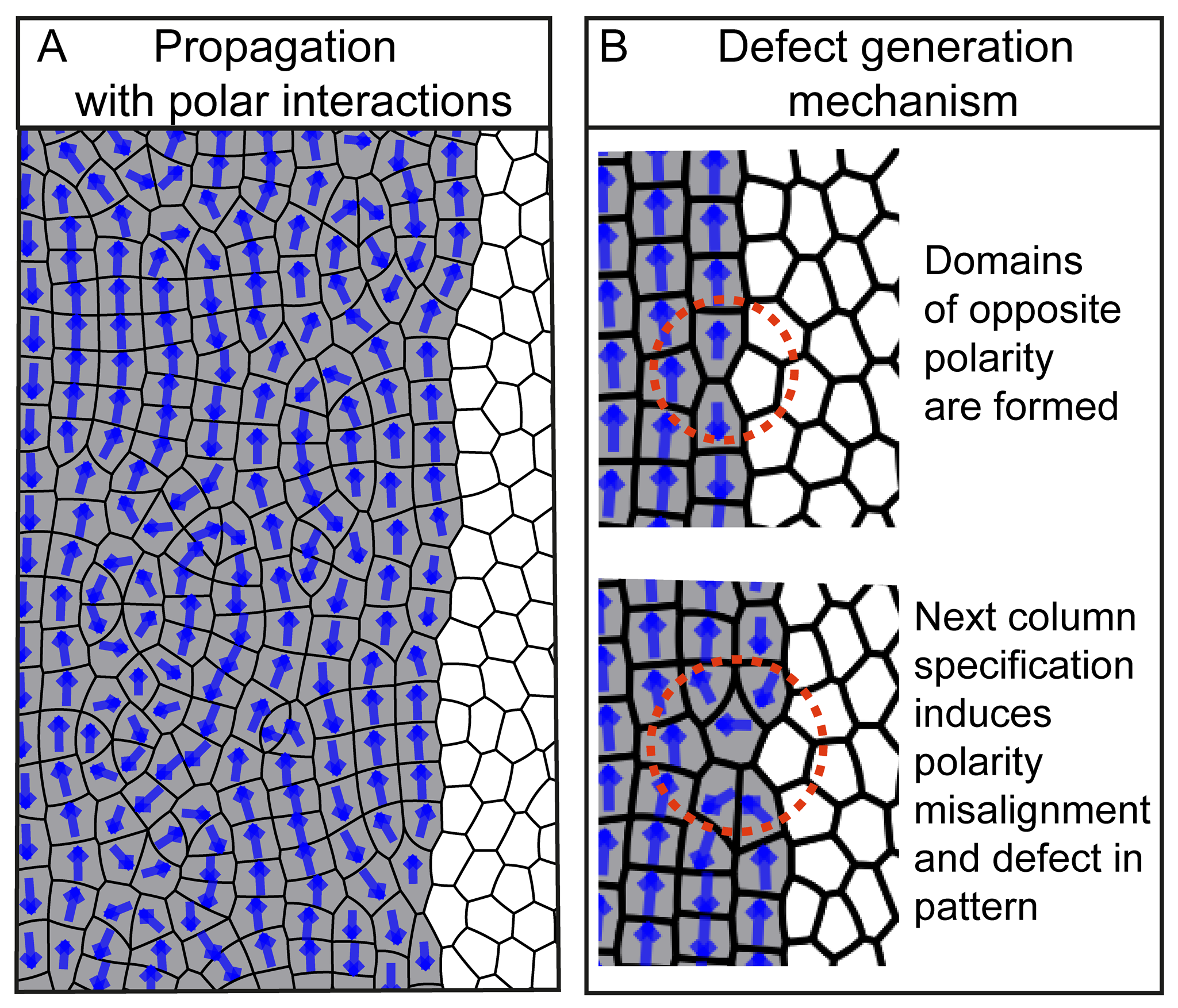

Supplement: Figure S7 — Model behavior with a polar PCP interaction. In this model variant, PCP parameters are chosen to favor apposition of A and B proteins across interfaces over A and A or B and B. (Parameters , , , , , , other parameters as described in the text). The simulation was started with one line of cells with a uniform polarity. Following this first line, each new row of cells is specified with a random distribution of polarity protein, and no global polarity cue is given. A) Example of simulation results: the cell packing does not show rectangular order. B) Snapshots of simulations illustrating the mechanism leading to patterning defects: polarity can point up or down, leading to domain formation. Because the resulting configuration is not favorable, the polarity reorients when the next column is specified, leading to polarity misalignment and eventually to a defect in cell packing. (TIF) [file pcbi.1002618.s007.tif]
