# Supplementary material for: Coupling Mechanical Deformations and Planar Cell Polarity to Create Regular Patterns in the Zebrafish Retina
Source: PLoS Comput Biol. 2012 Aug 23;8(8):e1002618. doi: 10.1371/journal.pcbi.1002618 (PMC3426565; doi:10.1371/journal.pcbi.1002618)
Supplement: Text S1 — Supplementary theoretical and computational methods. (PDF) [file pcbi.1002618.s009.pdf]

Salbreux *et al.*, Coupling Mechanical Deformations and Planar Cell Polarity to Create Regular Patterns in the Zebrafish Retina: Text S1

I. SUPPLEMENTARY METHODS

A. Dynamical equations

In this section we derive the explicit dynamical equations for the cell shapes and polarity protein concentrations. The dynamical equation for PCP can be rewritten from Eq. 6 in the main text as

$$\alpha_p\left(\frac{d}{dt}c_A^{\alpha,i}+\frac{c_A^{\alpha,i}}{l_i}\frac{dl_i}{dt}\right) = -\epsilon_{ab}c_B^{\alpha,i}+\epsilon_0 c_A^{\alpha,i}+\epsilon_1 c_B^{\beta,i}+\epsilon_2 c_A^{\beta,i} + \sum_{<j,i>}\left[-\frac{J_1}{l_i}(c_A^{\alpha,i}-c_A^{\alpha,j})+\frac{J_2}{l_i}(c_A^{\alpha,i}-c_B^{\alpha,j})\right]-T_e\log c_A^{\alpha,i}-\lambda_A^{\alpha} \tag{S1}$$

$$\lambda_A^{\alpha} = \frac{1}{\sum_{i\in\alpha}l_i}\left[-\epsilon_{ab}\sum_{i\in\alpha}l_i c_B^{\alpha,i}+\epsilon_0\sum_{i\in\alpha}l_i c_A^{\alpha,i}+\epsilon_1\sum_{i\in\alpha}l_i c_B^{\beta,i}+\epsilon_2\sum_{i\in\alpha}l_i c_A^{\beta,i} +2J_2\sum_{i\in\alpha}(c_A^{\alpha,i}-c_B^{\alpha,i})-T_e\sum_{i\in\alpha}l_i\ln c_A^{\alpha,i}\right], \tag{S2}$$

where β refers to the cell sharing the edge i with cell α, and $c_B^{\alpha,i}$ satisfies a symmetric equation where A and B are exchanged. λ_A^{α} is a Lagrange multiplier constraining the total number of proteins $\sum l_i c_A^{\alpha,i}$ within one cell. The main influence of cell shape, besides terms arising from protein number conservation, arises from the couplings J_1 and J_2 between neighboring edges: J_1 and J_2 have indeed dimensions of length and can be thought of as the typical length over which proteins interact on neighboring edges. In this description longer edges are therefore less sensitive to the concentration of their neighbors.

In addition, we assume the following form for the relaxation of the positions of the vertices and curvatures of the edges

$$\alpha_m\frac{d\mathbf{x}_k}{dt} = \sum_{\substack{\text{edges } i\\ \text{connected to } k}}\left[\gamma_0-\gamma_c\left(c_A^{\alpha_i,i}+c_A^{\beta_i,i}+c_B^{\alpha_i,i}+c_B^{\beta_i,i}\right)\right]\mathbf{e}_i^k \tag{S3}$$

$$\alpha_m\frac{dh_i}{dt} = -\frac{2}{3}l_i\left[\frac{\gamma_i}{R_i}-(P_{\beta_i}-P_{\alpha_i})\right] \tag{S4}$$

where :

- $\mathbf{e}_i^k$ is the unit vector tangent to edge i at vertex k and pointing outwards from the vertex.
- as described in the main text, h_i is the distance between the midpoints of the curved edge i and of the straight line joining its two endpoints. α_i and β_i are the two cells separated by the edge i, with the assumption that $h_i > 0$ if the curved edge bends towards cell α_i.
- R_i is the radius of curvature of edge i, related to h_i by $R_i = \frac{h_i^2+l_i^{s\,2}/4}{2h_i}$ where l_i^s is the length of the straight line joining the ends of the edge i
- $P_\eta = -K(A_\eta - A_0)$ is the (two-dimensional) pressure in cell η.

It is clear from Eqs. ?? and ?? that, in the steady state, force balance at the vertices is satisfied and the edges are circular arcs satisfying Laplace's law, $\Delta P = \frac{\gamma}{R}$.

B. Curvature corrections

Our model approximates regions of the retinal epithelium as flat, planar domains. In reality, however, the retina is a roughly hemispherical sheet. In this section, we show that our approximation is justified in the framework of the vertex model provided that the retina's radius of curvature R is sufficiently large, and we verify that this is the case for our system.

The radius of curvature R can be compared to two other length scales in the model: The typical size l of a single cell and the linear size w of the entire simulation domain. In the case of the zebrafish retina, $l \sim 5\mu$m, and the radius of the eye at 1mpf lies between 500μm and 1000μm, so that $l/R \lesssim 0.01$. Our simulations are run on domains typically extending over about 75μm, which implies $w/R \lesssim 0.15$. The major changes in cell packing at the retinal margin occur over an even smaller band, about 3 cells wide; if we take this distance to be the relevant measure of domain size, then $w/R \lesssim 0.03$. Below, we show that the leading corrections to the vertex model associated with curvature are of two sorts: Corrections associated with the projection of force vectors onto the plane tangent to the sphere at a given vertex and with the differences in length associated with following the curved sphere surface are of order l/R or smaller. In addition, although the surface of the sphere is locally planar, any mapping of a finite-sized domain of the sphere's surface to a plane necessarily introduces deformations whose magnitude increases with domain size. We find that corrections to the forces on vertices due to these deformations are of order $(w/R)^2$. Thus, neither correction exceeds about 2%.

To illustrate our point in more detail, we consider a vertex model representing a cell packing analogous to the model we have described, except that points are constrained to lie on a sphere of radius R. For the sake of simplicity we further assume that edges connecting two vertices are straight lines in 3D; models which include edge curvature introduce additional terms of order at most l/R. To compare this model to a planar model, we need an explicit mapping between the two. Here, we choose a mapping in which distortion is minimal near the equator and becomes progressively larger at one moves towards the poles. It is thus particularly well-suited to simulations that focus on a long strip near the equator—or, in our case, near the rim of a hemispherical retina. The coordinates of a point lying on a sphere are the polar angle θ, taken as 0 on the equator and the azimuthal angle ϕ (Fig. ??). These coordinates are mapped onto the Cartesian coordinates (x, y) in the plane according to the coordinate transformation $(x = R\theta, y = R\phi)$.

The length of the straight line joining two points A (θ_a, ϕ_a) and B (θ_b, ϕ_b) on a sphere is

$$l_{ab} = R\sqrt{(\cos\theta_a\cos\phi_a - \cos\theta_b\cos\phi_b)^2 + (\cos\theta_a\sin\phi_a - \cos\theta_b\sin\phi_b)^2 + (\sin\theta_a - \sin\theta_b)^2}\ . \tag{S5}$$

In the limit $l_{ab} \ll R$, we can write $\theta_b = \theta_a + \Delta\theta$ and $\phi_b = \phi_a + \Delta\phi$ and expand for small $\Delta\theta$ and $\Delta\phi$. We find, keeping terms up to third order, that

$$l_{ab} = R\sqrt{\Delta\theta^2 + \Delta\phi^2\cos^2\theta_a - \Delta\phi^2\Delta\theta\cos\theta_a\sin\theta_a}\ , \tag{S6}$$

or, in terms of the coordinates for the planar representation,

$$l_{ab} = \sqrt{(x_b - x_a)^2 + (y_b - y_a)^2 - \sin^2\left(\frac{x_a}{R}\right)(y_b - y_a)^2 - (y_b - y_a)^2(x_b - x_a)\cos\theta_a\sin\theta_a/R} \tag{S7}$$

This expression differs from the length of the straight line joining A and B in the planar representation, $l_{ab}^{planar} = \sqrt{((x_b - x_a)^2 + (y_b - y_a)^2}$, by a factor proportional to $\sin^2\left(\frac{x_a}{R}\right)$ at lowest order. The force exerted on vertex A by the tension γ on the edge joining A and B then has components given in terms of the (x, y) planar coordinates,

$$f_x = -\frac{\partial(\gamma l)}{\partial x_a} \simeq \gamma\left[\frac{x_b - x_a}{l} - \frac{l}{R}\frac{(y_b - y_a)^2}{4l^2}\sin\frac{2x_a}{R}\right] \tag{S8}$$

$$f_y = -\frac{\partial(\gamma l)}{\partial y_a} \simeq \gamma\left[\frac{y_b - y_a}{l} - \sin^2\left(\frac{x_a}{R}\right)\frac{y_b - y_a}{l} - \frac{l}{R}\frac{(y_b - y_a)(x_b - x_a)}{2l^2}\sin\frac{2x_a}{R}\right]\ . \tag{S9}$$

The first terms in brackets in the expressions for each force component are exactly the x and y components of a unit vector pointing from A to B, and thus correspond to the expression used in a planar vertex model (Eq. 4 in the main text). The additional terms represent curvature corrections that are neglected when computing forces in the planar vertex models. These corrections are of two types:

- The second term in Eq. **??** introduces a correction proportional to $\sin^2\left(\frac{x_a}{R}\right) \simeq (x_a/R)^2$. This correction is due to the deformation generated by the mapping from spherical to planar coordinates, which, for our choice of mapping, induces an increasing stretching in the y direction as one goes away from the equator of the sphere. If our simulations occur in a band of width w near the retinal margin, then x_a is at most w, so these corrections go like $(w/R)^2$, which is about 0.02 for the values of w and R given above.
- Corrections smaller than the main term by a factor l/R—or, in fact, of order lw/R^2 for this particular mapping—are due to effects associated with the curvature at the scale of a single cell. As noted above, in the case of the zebrafish retina epithelium, $\frac{l}{R} \sim 0.01$ so that these corrections are less than 1%.

One can verify that the forces associated with 2D pressures and area changes introduce similar corrections. Taken together, this analysis shows that the planar model gives correct forces near the margin of the eye with errors of about 2% or smaller. Changes in forces of this size will introduce an equivalent error in the position of the vertices; we do not expect such a small change to modify our basic conclusions.

C. Numerical methods

To simulate the polarity protein model, we have implemented an implicit-explicit scheme (IMEX scheme, [**?**]). This procedure was required due to the stiffness introduced by the logarithmic, entropic terms in F_p. To represent each concentration we introduce the auxiliary variable $X^{\alpha,i} = -\log(l_i c^{\alpha,i})$, where we have suppressed the subscript A or B. The entropic term ensures that on every edge the total quantity of protein $l \times c$ is greater than 0, so $X^{\alpha,i}$ remains real. The use of the auxiliary variable allows us to keep track of very small densities that would otherwise be below the machine precision.

The dynamical equation for the concentration of polarity proteins (Eq. 6 in the main text) can be rewritten in the compact form

$$\frac{dc_i}{dt} + \frac{c_i}{li}\frac{dl_i}{dt} = \sum_j U_{ij}c_j - T_e V_{ij}\log c_j \tag{S10}$$

where $(c_i)_{i=1..4N_e}$ denotes the vector of densities of the PCP proteins A and B on the two sides of the N_e edges in the packing, and U and V are two matrices of size $4N_e \times 4N_e$ whose coefficients can be determined from Eq. 6 in the main text. Only concentrations within the same cell are coupled by logarithmic terms, so, provided that the densities from the same cell are grouped together in the vector (c_i), V is banded, but U is typically dense. (V would in fact be diagonal, except that the Lagrange multiplier, Eq. **??**, couples the logarithm of concentrations on different edges.) The logarithmic term is the source of stiffness in the problem, and we therefore treat it implicitly in the simulations, while the first term is treated explicitly. This treatment allows us to avoid inverting the dense matrix U. Rewriting Eq. **??** for the variable X, we obtain

$$\exp(-X_i)\frac{dX_i}{dt} = -\sum_j [U_{ij}\frac{l_i}{l_j}\exp(-X_j) + l_i T_e V_{ij} X_j + l_i T_e V_{ij}\log l_j] \tag{S11}$$

which we simulate using the following IMEX scheme:

$$\sum_j \left[\frac{\exp(-X_i^n)}{\Delta t}\delta_{ij} + l_i T_e V_{ij}\right] X_j^{n+1} = \exp(-X_i^n)\frac{X_i^n}{\Delta t} - \sum_j \left[\frac{l_i}{l_j}U_{ij}\exp(-X_j^n) + l_i T_e V_{ij}\log l_j\right] , \tag{S12}$$

where n indexes successive time points spaced by Δt, and δ_{ij} is the Kronecker delta. The matrix on the left hand side is block diagonal, with one block corresponding to one cell. The block for cell α has the form

$$\frac{\exp(-X_i^n)}{\Delta t}\delta_{ij} + l_i T_e V_{ij}^\alpha = \left[\frac{\exp(-X_i^n)}{\Delta t} + l_i T_e\right]\delta_{ij} - T_e\frac{l_i l_j}{\sum_{k\in\alpha} l_k} \tag{S13}$$

Using the Sherman-Morrison inversion formula [?], it is straightforward to obtain the following expression for the inverse matrix

$$\left[\frac{\exp(-X_i^n)}{\Delta t}\delta_{ij} + l_i T_e V_{ij}^\alpha\right]^{-1} = \frac{1/l_i}{c_i/\Delta t + T_e}\delta_{ij} + \frac{1}{\frac{1}{T_e}\sum_{k\in\alpha} l_k - \sum_{k\in\alpha}\frac{l_k}{c_k/\Delta t + T_e}}\frac{1}{(c_i/\Delta t + T_e)(c_j/\Delta t + T_e)}$$

which, together with Eq. **??**, yields an analytical expression for X_i^{n+1} as a function of X_i^n. In addition, we renormalize the total number of proteins within each cell every 100 iterations to correct for additive numerical errors.

The dynamical equations for the vertex positions and edge curvatures are integrated with an explicit Euler scheme. The whole system is simulated with an adaptative stepsize method, with an absolute tolerance $\epsilon_{abs} = 0.01$ on the cell shape variables and absolute and relative tolerances $\epsilon_{abs} = \epsilon_{rel} = 0.001$ on the polarity protein variables.

D. Calculation of order parameters

1. *Nematic order parameter from the distribution of polarity proteins*

We detail here how we obtain a 2D nematic order parameter for the distribution of polarity proteins in a simulated cell packing. This order parameter determines the length and orientation of the bars indicating cell polarity in Fig. 5 of the main text and similar supporting figures and videos. A polar vector associated to the distribution of polarity proteins within cell α $\mathbf{P}^\alpha = (P_x^\alpha, P_y^\alpha)$ can be computed using the following expressions:

$$P_x^\alpha = \frac{1}{2}\left[\frac{\int_0^{2\pi} c_A^\alpha(\theta)\cos\theta d\theta}{\int_0^{2\pi} c_A^\alpha(\theta)d\theta} - \frac{\int_0^{2\pi} c_B^\alpha(\theta)\cos\theta d\theta}{\int_0^{2\pi} c_B^\alpha(\theta)d\theta}\right] \tag{S14}$$

$$P_y^\alpha = \frac{1}{2}\left[\frac{\int_0^{2\pi} c_A^\alpha(\theta)\sin\theta d\theta}{\int_0^{2\pi} c_A^\alpha(\theta)d\theta} - \frac{\int_0^{2\pi} c_B^\alpha(\theta)\sin\theta d\theta}{\int_0^{2\pi} c_B^\alpha(\theta)d\theta}\right] \tag{S15}$$

where θ refers to the angle relative to the horizontal axis, with the origin taken at the cell centroid. The global order parameter within the packing can then by obtained by averaging within each cell:

$$\mathbf{P} =< \mathbf{P}_\alpha > \tag{S16}$$

where the averaging is on cells α in the packing. For simulations with the parameter choice $\epsilon_1 = \epsilon_2$ however, the global polarity vanishes since head to head, tail to tail and head to tail configurations of polarity between neighbouring cells are equally favored. In that case, the global emergent order is characterized by a nematic order parameter. Introducing the notation $\mathbf{P}^\alpha = P^\alpha(\cos(\theta^\alpha), \sin(\theta^\alpha))$, the nematic order parameter is a 2D tensor with 2 independent parameters

$$Q = \begin{pmatrix} Q_{xx} & Q_{xy} \\ Q_{xy} & Q_{yy} \end{pmatrix} = \frac{1}{2}\begin{pmatrix} < \cos 2\theta^\alpha > & < \sin 2\theta^\alpha > \\ < \sin 2\theta^\alpha > & - < \cos 2\theta^\alpha > \end{pmatrix} = S_2 \begin{pmatrix} u_x^2 - \frac{1}{2} & u_x u_y \\ u_x u_y & u_y^2 - \frac{1}{2} \end{pmatrix} \tag{S17}$$

where in the last matrix, $S_2 = 2\sqrt{\mathrm{Tr}(Q^2)} = 2\sqrt{Q_{xx}^2 + Q_{xy}^2}$ is the order parameter satisfying $0 < S_2 < 1$, and (u_x, u_y) are the coordinates of the unit vector giving the global nematic orientation.

2. *Q_4 order parameter*

We detail here how we extract a fourfold symmetric order parameter Q_4 from the crosses that are fitted within each cell according to the procedure described in the text. As for the nematic order parameter, the four-fold order parameter can be viewed as a symmetric, traceless second rank tensor. (Strictly speaking, one might

expect fourfold rotational order to correspond to a fourth rank tensor. In two spatial dimensions, however, all real representations of the rotation group have the same dimension, and we may choose to express the fourfold rotational order in terms of a second rank tensor, thus taking advantage of what we already know about nematic order parameters.) The matrix is defined by the following expression, where θ_c^α now refers to the angle of one arm of the cross fitted to cell α

$$\begin{aligned}
\mathbf{Q}_4 &= \begin{pmatrix} Q_{xx}^4 & Q_{xy}^4 \\ Q_{xy}^4 & Q_{yy}^4 \end{pmatrix} = \begin{pmatrix} < \cos 4\theta_c^\alpha > & < \sin 4\theta_c^\alpha > \\ < \sin 4\theta_c^\alpha > & - < \cos 4\theta_c^\alpha > \end{pmatrix} \\
&= \begin{pmatrix} 8 < \cos^4 \theta_c^\alpha > -8 < \cos^2 \theta_c^\alpha > +1 & < 4\cos\theta_c^\alpha \sin\theta_c^\alpha (2\cos^2\theta_c^\alpha - 1) > \\ < 4\cos\theta_c^\alpha \sin\theta_c^\alpha (2\cos^2\theta_c^\alpha - 1) & -(8 < \sin^4\theta_c^\alpha > -8 < \sin^2\theta_c^\alpha > +1) \end{pmatrix} \\
&= Q_4 \begin{pmatrix} 8{v_x}^4 - 8v_x^2 + 1 & 4v_x v_y (2v_x^2 - 1) \\ 4v_x v_y (2v_x^2 - 1) & -(8v_y^4 - 8v_y^2 + 1) \end{pmatrix}
\end{aligned} \tag{S18}$$

where $Q_4 = \sqrt{\mathrm{Tr}(\mathbf{Q}_4^2)} = \sqrt{(Q_{xx}^4)^2 + (Q_{xy}^4)^2}$ is the magnitude of the fourfold order parameter and (v_x^4, v_y^4) are the coordinates of a unit vector giving the global fourfold orientation. The average is taken over different cells α. Finally, from those expressions the average angle $\theta_4 = \arctan \frac{v_y^4}{v_x^4}$ can be obtained

$$\theta_4 = \epsilon_2 \frac{\mathrm{sign}(Q_{xy}^4)}{2} \arccos \left(\epsilon_2 \sqrt{\frac{1}{2}\left(1 + \frac{Q_{xx}^4}{Q_4}\right)} \right) \tag{S19}$$

where ϵ_2 can be taken as 1 or -1 since the definition of θ_4 is invariant by rotation of $\frac{\pi}{2}$.

3. Positional order parameter

In order to obtain an order parameter for the positional order of the cells, we compute the Fourier transform of the density of cell centroids $n(\mathbf{x}) = \sum_{\text{cells } \alpha} \delta(\mathbf{x} - \mathbf{x}_\alpha)$, with $\mathbf{x}_\alpha$ the centroid of cell α, and normalize by the total number of cells N:

$$\rho(\mathbf{q}) = \frac{1}{N} \int d\mathbf{q}\, n(\mathbf{x}) e^{-i\mathbf{q}\mathbf{x}} \tag{S20}$$

$$= \frac{1}{N} \sum_{\text{cells } \alpha} e^{-i\mathbf{q}\mathbf{x}_\alpha} \tag{S21}$$

The corresponding structure factor S is given by

$$S(\mathbf{q}) = |\rho(\mathbf{q})|^2 \tag{S22}$$

For a crystalline solid, the function S exhibits Bragg peaks located on the reciprocal lattice of the Bravais lattice. As shown in Fig. **??**, a lattice consisting of points periodically distributed along one direction and disordered along the perpendicular direction has a structure factor exhibiting peaks in one direction and straight parallel lines in the other direction. Biological images of adult retina exhibit structure factors in between those two limits, with bright peaks in one direction corresponding to the column ordering and less pronounced peaks in the perpendicular direction, corresponding to the less pronounced correlations between the positions of individual cells in different columns (Fig. **??**). As the formation of an ordered column is the clearest feature of the neural retina pattern, we have chosen the amplitude of the peak with the smallest q in the direction of the column as the order parameter:

$$\rho_m = |\rho(\mathbf{q} = \mathbf{q}_0)| \tag{S23}$$

where q_0 is chosen as the lowest non-zero value of $\mathbf{q}$ maximizing the structure factor. In practice this value always corresponded to order along the direction of retinal growth and the formation of columns. It is also this

ordering that is most clearly reproduced by the simulations (Fig. ??).

[] U. M. Ascher, S. J. Ruuth, and B. T. R. Wetton, SIAM J Numer Anal **32**, 797 (1995).
[] M. Bartlett, Ann Math Stat **22**, 107 (1951).

II. SUPPORTING VIDEOS

- Video S1: Simulation of the progressive growth of a cone photoreceptor mosaic similar to that observed near the retinal margin in adult, wildtype fish; the cone fate is induced and the PCP pathway is activated in successive columns of cells at regular intervals, as described in the text.
- Video S2: Simulation of the relaxation of a cell packing with polarity proteins influencing edge tensions, under a global isotropic stress.
- Video S3: Simulation of the relaxation of a cell packing with polarity proteins influencing edge tensions, under a global anisotropic stress (the packing is compressed along the horizontal direction).
- Video S4: Simulation of the progressive growth of a retinal cone mosaic, but with polar rather than nematic interactions between polarity proteins.

III. SUPPORTING FIGURES

FIG. S1: **Identification of cell profiles at the apical surface of the adult zebrafish retina.** A–C) Retinal flat-mount from a young adult transgenic zebrafish (*ucd1*) in which the cone transducin alpha promoter drives expression of the EGFP reporter. A, C) All cones are green. B, C) cell boundaries at the level of the OLM are labeled with ZO-1 (white). B) UV cones (magenta stars) can be recognized by their large apical profiles relative to the other cones and rods. Red-green double cone pairs within a vertical column are tightly apposed with flattened interfaces (white arrows in B and also in E and H). D–F) Retinal flat-mount from a young adult transgenic zebrafish (*kj2*) in which the rod opsin promoter drives expression of the EGFP reporter. D, F) All rods are green. E, F) Cell boundaries at the level of the OLM are labeled with ZO-1 (white). F) The initial rods insert between vertical cone columns at the intersections between red, green, blue and UV cones in adjacent columns. G–I) Retinal flat-mount from an older adult rod transgenic zebrafish (*kj2*). Rods continue to accumulate between adjacent vertical cone columns. J–L) Flat-mount at the margin of the retina from a young adult transgenic zebrafish (*mi2002*) in which the promoter from the glial-specific gene, *gfap*, drives expression of the EGFP reporter in Müller glial cells. The germinal zone is at the right. J, L) Müller glial cells at the apical surface (green); thin lamellar processes completely surround all rod and cone photoreceptors at the OLM, and Müller glia also account for the irregular profiles in the germinal zone and adjacent region where cones are differentiating (magenta arrows in K and L; also see Fig. 3A).

FIG. S2: **Example of the segmentation procedure.** A) Original image, B) Segmentation result, C) Overlay (original image has been darkened and segmentation lines thickened for visualization purposes).

FIG. S3: **Fourier transform of cell centroid positions.** Top row: examples of Fourier transform for points distributed randomly, periodic columns with random relative shifts, and points distributed on a square lattice. Middle row: simulation results for different values of the parameter γ_c. Lower row: experimental data.

FIG. S4: **Differentiation of cone photoreceptors and elaboration of the apical process.** A) Retinal cryosection from larval transgenic (*mi2009*) fish at 4 days post-fertilization (dpf), in which blue and UV cones express the reporters mCherry and EGFP (pseudocolored blue and magenta, respectively), which fill the cytoplasm of the cells. The OLM (arrow) is labeled by ZO-1 immunostaining (yellow). The developing inner and outer segments of the cones project apically beyond the OLM. B) Larval *mi2009* fish at 4 dpf immunolabeled for Crb2a (yellow). Note that the Crb2b protein extends beyond the OLM (arrow) on the plasma membrane of the inner segments. C) By 10 dpf, the cone inner and outer segments have elongated further and the Crb2a protein also extends further apically on the inner segments. The arrow indicates the level of the OLM. D) Differential interference contrast (DIC) image of a retinal cryosection from an adult zebrafish, immunolabeled with ZO-1 (yellow) to label the OLM (arrow). The inner and outer segments of cones extend apically; the UV cones are the shortest, the blue (B) cones are longer, and the red and green (RG) double cones are the longest. E) A DIC image with immunolocalization of Crb2a protein on the inner segments of the cones. The black arrow indicates the OLM. The inset shows the Crb2a protein at the interface between red and green double cones (white arrows). F) Same image as panel E without the DIC channel. G) Differential interference contrast (DIC) image of a retinal cryosection from an adult transgenic zebrafish (*mi2002*), expressing a fluorescent reporter in Müller glia (cyan), and immunolabeled with Crb2a (yellow). H) Same image as panel E without the DIC channel. The processes of Müller glia (white arrows) extend apically beyond the OLM, but not as far as the Crb2a (yellow). Müller processes do not separate the interface between the inner segments of red and green double cone pairs, which have strong staining for Crb2a (yellow arrows). I) Left half: DIC image of a retinal cryosection from an adult transgenic zebrafish (*mi2002*), expressing a fluorescent reporter in Müller glia (cyan), and immunostained with *zpr1*, which labels red and green cones (magenta). Right half: same image without the DIC channel. The processes of the Müller glia (white arrow) are not interposed between the inner segments of red and green double cone pairs (magenta arrow). J–L) Individual optical sections from a z-stack confocal image of Crb2a immunolabeling near the retinal margin in a retinal flat-mount (cropped version from the same image series shown in Figure 6A). Panel J is at the level of the OLM, panel K is the subapical region (SAR) at 2.5 μm from the OLM, and panel L is the SAR at 5 μm from the OLM. Identity of cone photoreceptor subtypes is indicated by asterisks: red, green, blue, and UV (magenta), respectively. In the SAR, Crb2a has a planar polarized distribution it is expressed at higher levels at the interfaces of red, green, and blue cones within a column. The retinal surface is curved, so the left and right sides of each panel are more apical than the center (and thus show a somewhat more polarized Crb2a distribution in J and K).

FIG. S5: **Model components.** A) Schematic of quadratic interaction in the polarity protein effective energy F_p. Only interactions involving the concentration $c_A^{\alpha,i}$ are represented. B) Redistribution of polarity proteins in topological transitions. Left: shrinking of an edge to a 4-fold vertex; the polarity proteins on the vanishing edge are redistributed onto neighboring edges. Right: formation of a new edge from a 4-fold vertex; a minimal amount of polarity proteins is redistributed onto the newly formed edge. C) Schematic illustrating how cells adopt a rectangular shape in the simulations. Left: with uniform tension, cells typically adopt hexagonal shapes where edges meet at vertices with 120° angles. Right: when the polarity protein dynamics is turned on, polarity proteins accumulate on opposite edges of cells, which results in an decrease of edge tension on 2 opposite sides and an increase in tension on the other 4 sides. To satisfy force balance, the cells must then deform their shapes towards a nearly rectangular shape. When minimum and maximum tensions in a cell satisfy $\gamma_{min} << \gamma_{max}$, the cell shape is close to a rectangle and, in a perfectly regular packing, the length of the edges with polarity proteins is approximately $\frac{3l}{2}$, where l is the length of a side in the original hexagonal packing. Assuming each polarity protein is concentrated on one of two opposite edges in the cell, the concentration reached is about $c_A = \frac{2}{3l}, c_B = 0$ or $c_B = \frac{2}{3l}, c_A = 0$. Counting the polarity proteins on both sides of the edges, this results in the estimates of γ_{min} and γ_{max} indicated below the schematic.

FIG. S6: **Robustness analysis of the formation of a regular packing in our simulations.** Columns, from left to right: nematic order parameter from the distribution of PCP proteins; solid order parameter obtained from the Fourier transform of the cell centroid positions; Q_4 order parameter; examples of the pattern produced for extreme values of the parameters. The red line in the middle column indicates a rough empirical criterion ($\rho_m > 0.65$) for the order parameter values above which our simulations show an ordered rectangular packing without defects resembling the experimental pattern. Black points correspond to parameters for simulations presented in the text. Values were obtained from 3 simulations with 500 cells for each point; error bars = SEM.

FIG. S7: **Model behavior with a polar PCP interaction.** In this model variant, PCP parameters are chosen to favor apposition of A and B proteins across interfaces over A and A or B and B. (Parameters $\epsilon_2/\epsilon_{ab} = 0$, $\epsilon_1/\epsilon_{ab} = 2$, $\epsilon_0/\epsilon_{ab} = -0.4$, $J_1/l\epsilon_{ab} = 0.24$, $J_2/l\epsilon_{ab} = 0.12$, $T_e l/\epsilon_{ab} = 0.03$, other parameters as described in the text). The simulation was started with one line of cells with a uniform polarity. Following this first line, each new row of cells is specified with a random distribution of polarity protein, and no global polarity cue is given. A) Example of simulation results: the cell packing does not show rectangular order. B) Snapshots of simulations illustrating the mechanism leading to patterning defects: polarity can point up or down, leading to domain formation. Because the resulting configuration is not favorable, the polarity reorients when the next column is specified, leading to polarity misalignment and eventually to a defect in cell packing.

FIG. S8: **Mapping from a hemispherical to a planar surface.** For a hemisphere of radius R, Cartesian coordinates x, y in the plane are related to the polar and azimuthal angles θ and ϕ on the hemisphere as $x = R\theta$ and $y = R\phi$.
